# Different responses to DNA damage determine ageing differences between organs

**DOI:** 10.1111/acel.13562

**Published:** 2022-03-04

**Authors:** Maria Vougioukalaki, Joris Demmers, Wilbert P. Vermeij, Marjolein Baar, Serena Bruens, Aristea Magaraki, Ewart Kuijk, Myrthe Jager, Sarra Merzouk, Renata M.C. Brandt, Janneke Kouwenberg, Ruben van Boxtel, Edwin Cuppen, Joris Pothof, Jan H. J. Hoeijmakers

**Affiliations:** ^1^ Department Molecular Genetics Erasmus University Medical Center Rotterdam Rotterdam The Netherlands; ^2^ Princess Máxima Center for Pediatric Oncology Oncode Institute Utrecht The Netherlands; ^3^ Center for Molecular Medicine University Medical Center Utrecht Utrecht The Netherlands; ^4^ Department of Developmental Biology Oncode Institute Rotterdam The Netherlands; ^5^ Division Biomedical Genetics Center for Molecular Medicine and Cancer Genomics Netherlands University Medical Center Utrecht Utrecht University Utrecht The Netherlands; ^6^ Department of Genetics Center for Molecular Medicine University Medical Center Utrecht Utrecht University Utrecht The Netherlands; ^7^ Hartwig Medical Foundation Amsterdam Netherlands; ^8^ Institute for Genome Stability in Ageing and Disease Cologne Excellence Cluster for Cellular Stress Responses in Aging‐Associated Diseases (CECAD) University Hospital of Cologne Cologne Germany

**Keywords:** adult stem cells, DNA damage response, ERCC1, genome maintenance, liver, nucleotide excision repair, organoids, small intestine

## Abstract

Organs age differently, causing wide heterogeneity in multimorbidity, but underlying mechanisms are largely elusive. To investigate the basis of organ‐specific ageing, we utilized progeroid repair‐deficient *Ercc1^Δ^* ^/−^ mouse mutants and systematically compared at the tissue, stem cell and organoid level two organs representing ageing extremes. *Ercc1^Δ^* ^/−^ intestine shows hardly any accelerated ageing. Nevertheless, we found apoptosis and reduced numbers of intestinal stem cells (ISCs), but cell loss appears compensated by over‐proliferation. ISCs retain their organoid‐forming capacity, but organoids perform poorly in culture, compared with WT. Conversely, liver ages dramatically, even causing early death in *Ercc1*‐KO mice. Apoptosis, p21, polyploidization and proliferation of various (stem) cells were prominently elevated in *Ercc1^Δ^* ^/−^ liver and stem cell populations were either largely unaffected (Sox9+), or expanding (Lgr5+), but were functionally exhausted in organoid formation and development *in vitro*. Paradoxically, while intestine displays less ageing, repair in WT ISCs appears inferior to liver as shown by enhanced sensitivity to various DNA‐damaging agents, and lower lesion removal. Our findings reveal organ‐specific anti‐ageing strategies. Intestine, with short lifespan limiting time for damage accumulation and repair, favours apoptosis of damaged cells relying on ISC plasticity. Liver with low renewal rates depends more on repair pathways specifically protecting the transcribed compartment of the genome to promote sustained functionality and cell preservation. As shown before, the hematopoietic system with intermediate self‐renewal mainly invokes replication‐linked mechanisms, apoptosis and senescence. Hence, organs employ different genome maintenance strategies, explaining heterogeneity in organ ageing and the segmental nature of DNA‐repair‐deficient progerias.

## Graphical abstract

Organs age differently. To investigate the basis of organ‐specific ageing we systematically compared at the tissue, stem cell and organoid level two organs representing ageing extremes, from accelerated aged, repair‐deficient *Ercc1* *^Δ^* ^/−^ mouse mutants. Very short‐lived cells, such as those of intestinal epithelium, have low damage tolerance and opt primarily for apoptosis and cell replacement. On the other extreme, liver (and by inference also neuronal tissue) with long‐lived cells, invest in cell survival by damage sensing and repair primarily linked with the transcribed compartment. They also may have high damage tolerance and consequently higher senescence levels. The differences in genome maintenance strategies we reveal, explain heterogeneity in organ ageing and the segmental nature of DNA‐repair‐deficient progerias.

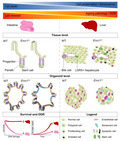

**Abbreviations**
DDR: DNA damage response
GG‐NER: Global genome nucleotide excision repair
HR: Homologous recombination
HSC: Hematopoietic stem cell
ICS: Intestinal stem cell
LCS: Liver stem cell
NHEJ: Non‐homologous end‐joining
SC: Stem cell
SI: Small intestine
TCR: Transcription‐coupled repair
TLS: Translesion synthesis
XLR: Cross‐link repair

## 1 INTRODUCTION

Accumulation of DNA damage is recognized as a principal cause of systemic ageing (Hoeijmakers, 2009; Niedernhofer et al., 2018; Schumacher et al., 2021). DNA lesions interfere with DNA function and activate an intricate DNA damage response (DDR), which triggers repair and decides on cell fate including cell cycle arrest (senescence), mutagenesis, premature differentiation or cell death. Since DNA is at the top of the informational hierarchy, genetic erosion has very diverse, lasting consequences, impairing cell and tissue functioning causing pathology and cancer (Marteijn et al., 2014; Vermeij, Dollé, et al., 2016; Vermeij, Hoeijmakers, & Pothof, 2016). However, how DNA damage shapes ageing and how this relates to organ/tissue‐specific ageing trajectories is largely unknown.

Adult stem cells (SC) gradually lose functionality with age (McNeely, 2020). Mouse models mimicking DNA‐repair‐deficient human syndromes, revealed numerous features of ageing pathology (Marteijn et al., 2014), including functional impairment of hematopoietic stem cells (HSCs) (Cho et al., 2013; Navarro et al., 2006; Nijnik et al., 2007; Prasher et al., 2005; Rossi et al., 2007; Walter et al., 2015). Also, damage likely compromises HSCs and progenitor functionality in wt mice and humans (Beerman et al., 2014; Flach et al., 2014; Rube et al., 2011). In telomerase‐deficient mice, intestinal stem cells (ISCs) and progenitors undergo apoptosis or cell cycle arrest (Begus‐Nahrmann et al., 2009; Sperka et al., 2011). Hair follicle stem cells prematurely differentiate in a mouse model of the human progeroid DNA repair syndrome trichothiodystrophy and upon exogenous damage, similarly to naturally aged mice (Matsumura et al., 2016). Muscle stem cells decline in number and function in *Ercc1*‐repair‐deficient progeroid mutants, as in aged mice (Alyodawi et al., 2019; Lavasani et al., 2012).

Here, we compared intestine and liver, and their stem cells, organoids and damage responses in the *Ercc1*‐deficient mouse model of the XFE human progeroid syndrome (Niedernhofer et al., 2006). ERCC1 is in a complex with XPF involved in damage excision in multiple DNA repair systems: global genome nucleotide excision repair (GG‐NER) and transcription‐coupled repair (TCR), which remove helix‐distorting and transcription‐stalling lesions, (deficient in the rare human genetic disorders xeroderma pigmentosum and Cockayne syndrome, respectively (de Laat et al., 1999; Marteijn et al., 2014)), as well as interstrand cross‐link repair (defective in Fanconi's anaemia) and single‐strand annealing repair of persistent double strand breaks (Ahmad et al., 2008; Gillet & Scharer, 2006; Kuraoka et al., 2000; Niedernhofer et al., 2004). Thus, *Ercc1^Δ^* ^/−^ mice carrying one hypomorphic truncation allele and one null allele, harbour defects in four repair systems and combine multiple human genome instability disorders. They have largely normal embryonal development, but show after birth numerous progressive progeroid symptoms (Dolle et al., 2011), strikingly similar to natural ageing, which limit lifespan to 4–6 months (Vermeij et al., 2016b). Using this model, we analysed at tissue, SCs and organoids levels liver and small intestine, which vastly differ in ageing features in order to comprehend why organs and tissues age differently.

## 2 RESULTS

### 2.1 Contrasting ageing physiology and checkpoints of small intestine and liver from *Ercc1^Δ^* ^/−^ mice

**FIGURE 1:**
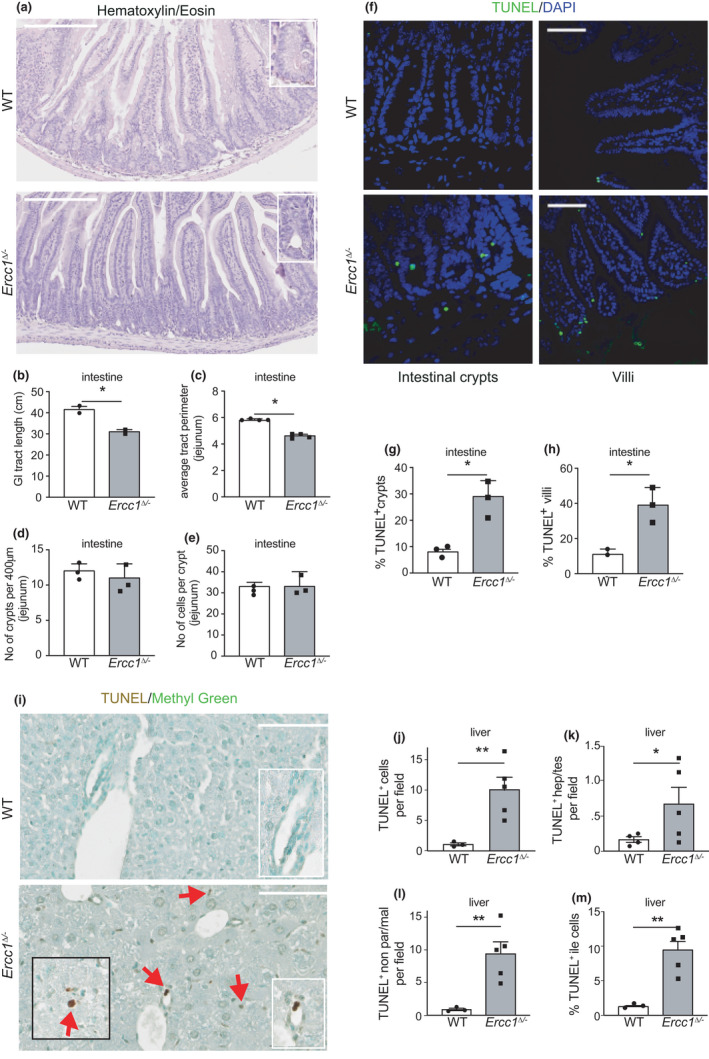
Aging‐related phenotypic features of small intestinal and liver from progeroid *Ercc1^Δ^* ^/−^ mice. (a) Intestinal tissue from 15‐week‐old *Ercc1^Δ^* ^/−^ and control mice stained with haematoxylin and eosin. Bars 200 μm. (b, c) Intestinal length (b, *n* = 2) and perimeter (c, *n* = 4) from 15‐week‐old mice of indicated genotypes. (d) Jejunal crypt density of 15‐week‐old wt and mutant mice. Crypts were counted on paraffin‐embedded 4μm slices of intestinal tissue. The number of crypts of progeroid *Ercc1^Δ^* ^/−^ mice is not significantly reduced in spite of the overall cachexia and decreased organ size, *p* = 0.7328 (*n* = 3). (e) Cell density in jejunal crypts of 15‐week‐old wt and *Ercc1^Δ^* ^/−^ mice. Cells were counted on DAPI stained 4μm intestinal tissue slices as in (d), *p* = 0.5415 (*n* = 3 mice). (f) Immunofluorescent images of small intestine crypt and villi from sections stained for apoptosis (TUNEL), counterstained with DAPI. Bars 50 μm. (g, h) Apoptosis index in crypts (g) and villi (h), (*n* = 3 mice). (i) Liver tissue from 15‐week‐old *Ercc1^Δ^* ^/−^ and wt mice assessed for apoptosis (TUNEL). Red arrows: TUNEL^+^ cells; black cut‐out depicts a TUNEL^+^ large hepatocyte. Bars 100 μm. (j–m) Apoptosis index in the liver, parenchymal, non‐parenchymal (l), and biliary cell (m) population of 15‐week‐old wt and mutant mice (*n* = at least 3 mice for WT, *n* = 5 mice for mutant groups). Quantification of TUNEL^+^ nuclei was performed on DAB‐stained liver sections. Data: mean ± SEM. *p < 0.05, **p < 0.01

While liver suffers dramatic ageing pathology in *Ercc1* ***^Δ^* ^/−^** mutants and in full *Ercc1*‐KO is lifespan limiting, small intestine (SI) seems unaffected (Dolle et al., 2011; Gregg et al., 2012; Wang et al., 2012). To compare these extremes in ageing, we choose the age of 15 weeks, when *Ercc1* ***^Δ^* ^/−^** mice manifest numerous ageing symptoms, without being moribund (Dolle et al., 2011). Histochemical evaluation of *Ercc1^Δ^* ^/−^ SI revealed no overt abnormalities (Figure 1a), aside from a smaller intestinal tract and perimeter (Figure 1b,c) in line with the reported cachexia of the mutant (Dolle et al., 2011) and smaller overall size of *Ercc1^Δ^* ^/−^ mice. The number and density of crypts appeared similar to WT (Figure 1d,e) and the ability of goblet cells to secrete mucus seems unchanged, suggesting normal intestinal function (Figure S1A). However, apoptosis—an outcome linked with ageing‐related homeostatic deregulation (Muradian & Schachtschabel, 2001) in naturally aged crypts (Martin et al., 1998) appeared significantly elevated with cells dying at the bottom and higher‐up in both *Ercc1^Δ^* ^/−^ villi and crypts. Hence, apoptosis is not restricted to a specific cellular compartment or stage of differentiation (Figure 1f–h), consistent with a stochastic origin and congruent with accelerated ageing.

Contrary to SI, *Ercc1^Δ^* ^/−^ liver is known to suffer from severe ageing pathology (Dolle et al., 2011; Gregg et al., 2012; Vermeij et al., 2016b; Weeda et al., 1997), confirmed for the *Lgr5^EGFP^Ercc1^Δ^* ^/−^ model used here in Figure S1B–E. TUNEL immunostaining revealed apoptosis to be clearly enhanced in *Ercc1^Δ^* ^/−^ liver (Figure 1i–j). Close morphological inspection revealed that nearly all cell types are affected (Figure 1k–m). In fact, the increase of TUNEL + hepatocytes was relatively modest, although, notably, apoptosis included also polykaryons, that is, the equivalent of many diploid hepatocytes. Moreover, hepatocytes expressing cyclin‐dependent kinase inhibitor p21 were ~40‐fold increased (Figure S2A–B) and a ~4‐fold increase in biliary cells was found (Figure S2C), consistent with the systemic nature of the repair defect and wide‐spread premature ageing features.

p21 expression in mice carrying (liver‐specific) DNA repair deficiency is correlated with senescence (Ogrodnik et al., 2017). Recently, Yousefzadeh and coworkers (Yousefzadeh et al., 2020) convincingly demonstrated extensive senescence in 10 organs of *Ercc1^Δ^* ^/−^ mice, including liver, starting at the age of 12 weeks, progressively increasing with time. We examined liver tissue of 15‐week‐old *Ercc1^Δ^* ^/−^ mice, but unexpectedly, despite multiple trials and positive controls (see below), did not detect significant loss of LaminB1 and nuclear HMGB1 immunosignals nor significantly increased IL‐6 expression that would suggest overt senescence, at this age in our *Ercc1^Δ^* ^/−^ mouse line (Figure S3A–F). Further investigation is warranted to find out whether differences in, for example housing conditions, play a role in the age of onset of senescence in liver, including food, for which *Ercc1^Δ^* ^/−^ mutants are extremely sensitive regarding accelerated ageing (Vermeij, Dollé, et al., 2016) and which likely influences anti‐oxidant buffering and DNA damage load (Milanese et al., 2019).

In conclusion, in this and other studies (Dolle et al., 2011; Weeda et al., 1997; Yousefzadeh et al., 2020), *Ercc1^Δ^* ^/−^ liver displays numerous accelerated ageing features, in hepatocytes and biliary cholangiocytes, in sharp contrast to SI, although elevated apoptosis in villi and the regenerative and amplifying compartments of crypts suggests altered homeostatic regulation in this organ as well.

### 2.2 Tissue‐specific regenerative responses parallel homeostatic deregulation and pathology in *Ercc1^Δ^* ^/−^ small intestine and liver

**FIGURE 2:**
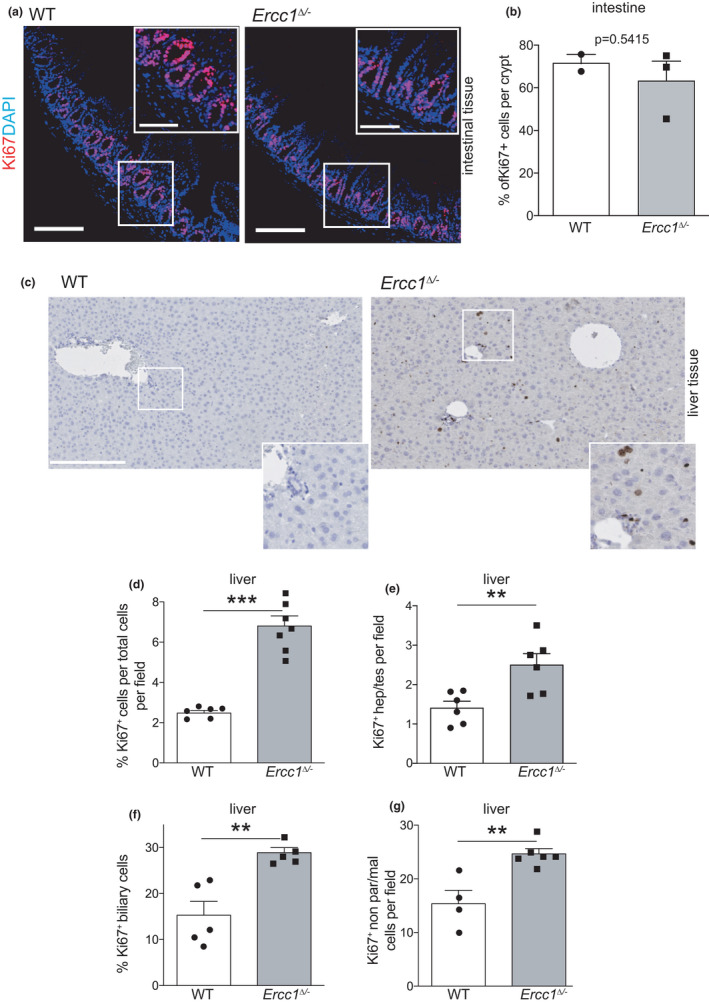
Steady‐state regenerative potential of intestine and liver of progeroid *Ercc1^Δ^* ^/−^ mice. (a) Immunofluorescence of intestinal tissue sections from 15‐week‐old wt and mutant mice stained for proliferation marker Ki67, nuclei counterstained with DAPI. Insets here and in other panels are digitally magnified images. Bars 10 μm (5 μm for insets). (b) Steady‐state proliferative index of small intestine from 15‐week‐old *Ercc1^Δ^* ^/−^ (*n* = 3) and control mice (*n* = 2). (c) Immunohistochemical staining for proliferation marker Ki67 of liver from 15‐week‐old *Lgr5^EGFP^Ercc1^Δ^* ^/−^ and wt mice. Nuclei counterstained with haematoxylin. Bars 250 μm. (d) Proliferative index of *Lgr5^EGFP^Ercc1^Δ^* ^/−^ liver. Quantitation of Ki67^+^ cells per total cells in a field as represented in (c) from 15‐week‐old mutant and wt mice. More than 5 fields were quantified from *n* = 7 mice per genotype. (e–g) Proliferative index of various cell populations (identified on morphology and location) in progeroid *Lgr5^EGFP^Ercc1^Δ^* ^/−^ and wt liver. Note that nearly all cell populations in *Ercc1^Δ^* ^/−^ liver show increased proliferation. Over 5 fields were quantified from at least 4 mice per genotype. Data: mean ± SEM, **p < 0.01, ***p < 0.001

Previous research provided evidence for limited regenerative proficiency of *Ercc1^Δ^* ^/−^ liver following partial hepatectomy (Gregg et al., 2012), but which cells are implicated and whether SI is affected as well is unknown. Ki67 immunostaining indicated that the proliferative index of *Ercc1^Δ^* ^/−^ intestinal crypts did not differ from controls (Figure 2a,b). In contrast, *Ercc1^Δ^* ^/−^ liver showed a prominent upregulation of Ki67^+^ cells in nearly all cell populations (Figure 2c–g). Thus, in progeroid *Ercc1^Δ^* ^/−^ mice SI displays normal regeneration rates, in spite of increased apoptotic events. However, compared with the very high cell turn‐over, cell loss is relatively small and compensatory over‐proliferation would likely go undetected. In contrast, in the liver, which normally has low turn‐over, homeostasis is severely affected, as for other NER‐deficient mouse mutants (Barnhoorn et al., 2014). Hence, DNA repair deficiency triggers cell loss in many cell types in liver, but also enhanced compensatory cell division.

### 2.3 Distinct responses of *Ercc1^Δ^* ^/−^ SC types to unrepaired damage

**FIGURE 3:**
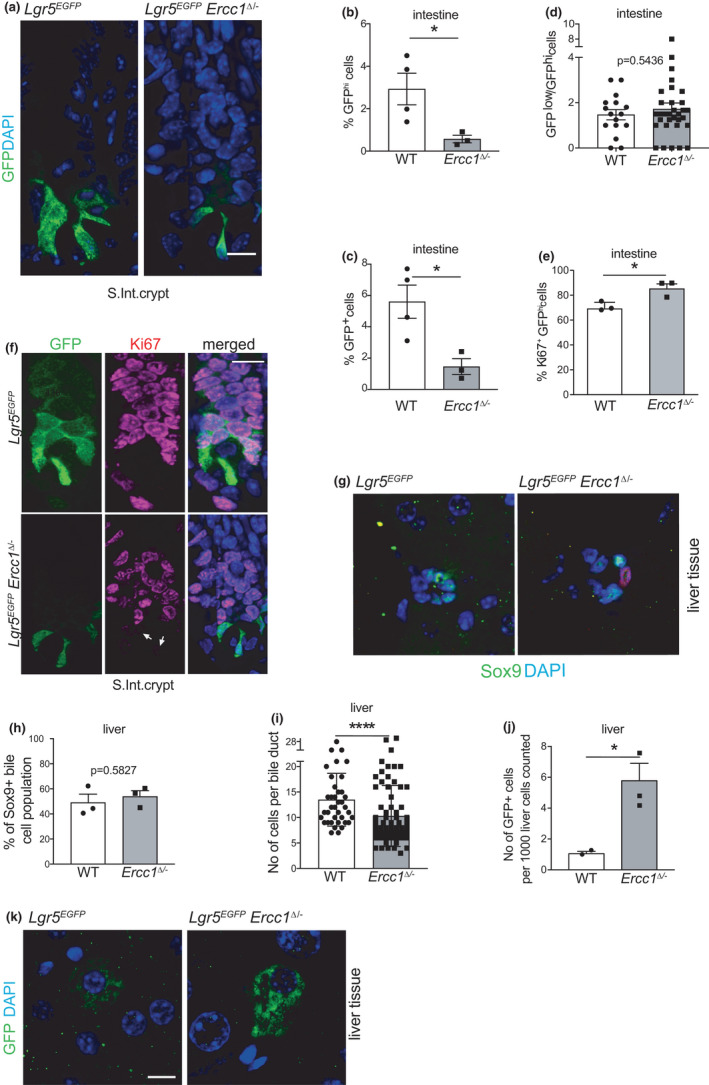
Characterization of small intestinal and liver stem cell populations in progeroid *Ercc1^Δ^* ^/−^ mice. (a) Enhanced green fluorescent protein (EGFP) immunostaining in crypts of the small intestine from 15‐week‐old mutant and wt mice. Nuclei counterstained with DAPI. Bars 10 μm. (b) Quantitation of intestinal crypt cells expressing high levels of GFP (GFP^hi^) by flow cytometry of 15‐week‐old wt (*n* = 4) and *Ercc1^Δ^* ^/−^ (*n* = 3) mice. (c) Quantitation of the total EGFP‐expressing (EGFP^+^) cell population in small intestinal crypts of wt (*n* = 4) and mutant (*n* = 3) mice by flow cytometry. (d) Ratio of crypt cells expressing low (EGFP^low^, i.e., downstream progenitor population) versus high levels of EGFP (GFP^hi^, i.e., stem cells) in the intestine of the indicated mice. Each column represents collectively the values for individual crypts for 3 mice per genotype. (e) Quantitation of the GFP^hi^ intestinal population that expresses Ki67 proliferation marker from immunofluorescently stained tissue of 15‐week‐old mice of the indicated genotypes (*n* = 3 mice per genotype). Note the increase of proliferative stem cells in progeroid *Ercc1* mutant mice. (f) Immunofluorescence images of paraffin‐embedded intestinal tissue from 15‐week‐old *Lgr5^EGFP^ Ercc1^Δ^* ^/−^ and wt mice, stained for EGFP and Ki67. Bars 10 μm. (g) Immunofluorescent staining for Sox9 expression (green) in bile duct cells of liver sections from 15‐week‐old *Lgr5^EGFP^ Ercc1^Δ^* ^/−^ and wt mice, along with Ki67 (red). Bar 10 μm. (h) Percentage of Sox9+ bile cell population per total cells in liver ducts of 15‐week‐old *Ercc1^Δ^* ^/−^ and wt mice, quantified from 4μm liver sections stained as in (g) (*n* = 3 mice). (i) Average number of cells per bile duct in livers of 15‐week‐old *Ercc1^Δ^* ^/−^ and wt mice, quantified from 4 μm liver sections. Data represent means with SD from quantitation of cells in round bile ducts from 3 mice per genotype and at least 10 bile ducts per mouse. (j) EGFP‐expressing cells in the liver of *Lgr5^EGFP^Ercc1^Δ^* ^/−^ (*n* = 3) and control (*n* = 2) mice. (k) EGFP + Lgr5‐marked stem cells in liver of 15‐week‐old *Lgr5^EGFP^Ercc1^Δ^* ^/−^ and control mice stained for GFP. Bars 10 μm. Data: mean ± SEM unless otherwise specified. *p < 0.05, ****p < 0.0001

To further investigate the fate of SCs, we focused on *Lgr5* ^+^ stem cells responsible for steady‐state intestinal homeostasis but also damage‐induced liver regeneration (Barker et al., 2007; Huch et al., 2013). We crossed *Lgr5^EGFP^* ^−^ *^ires^* ^−^ *^CreERT2^* (Barker et al., 2007) and *Ercc1^Δ^* ^/−^ mice and monitored EGFP‐expressing (EGFP^+^) cells in 15‐week‐old *Lgr5^EGFP^Ercc1^Δ^* ^/−^ SI and liver. Flow cytometry revealed a strikingly reduced number of high EGFP‐expressing (EGFP^hi^) ISCs in progeroid mice (Figure 3a,b). Immunohistology and flow cytometry disclosed also erosion of the total EGFP^+^ population in mutant intestine (Figure 3c). Unaltered ratios of EGFP^low^ to EGFP^hi^ cells (Figure 3d) in crypts and seemingly unaffected intestinal functionality (Figure S1A) suggest that ICSs in progeroid mice are eliminated randomly through apoptosis (as shown in Figure 1g,h), rather than premature differentiation. To examine how *Ercc1^Δ^* ^/−^ intestine maintains homeostasis, given the increased apoptosis and reduced numbers of SCs, we analysed SI sections for co‐occurrence of Ki67 and EGFP expression and found that mutant crypts bear a higher percentage of Ki67+ EGFP^hi^ SCs and progenitors indicating that they are more in cycle than crypts of *Lgr5^EGFP^* control mice (Figure 3e,f).

A liver cell type, attributed with SC properties, is the Sox9+ cholangiocyte (Gilgenkrantz & Collin de l'Hortet, 2018). Close examination of bile ducts revealed unaltered Sox9^+^ cell pools (Figure 3h), but increased bile cell nuclear size (Figure S1c,d) and a more disorganized structure of the ducts in *Ercc1^Δ^* ^/−^ liver (Figure 3g). In addition, the number of cells per bile duct was significantly reduced (Figure 3i), in line with the evidence for apoptosis of this cell population in mutant liver (Figure 1m). Apparently, cell loss is not adequately compensated by increased cell division leading to aberrant duct morphology and possibly function.

To further investigate the impact of *Ercc1* deficiency on liver stem cells (LSCs), we quantified *Lgr5^EGFP^* ^+^ cells on paraffin‐embedded liver sections. EGFP^+^ mutant SCs appeared more abundant and display intenser GFP fluorescence than in WT (Figure 3j,k). A prominent characteristic of *Ercc1^Δ^* ^/−^ liver is the abundance of large polyploid nuclei (McWhir et al., 1993; Weeda et al., 1997). We found that a significant fraction of *Ercc1^Δ^* ^/−^ *Lgr5^EGFP^* ^+^ cells has enlarged nuclear size. But despite some EGFP^+^ cells in mutant mice looking polyploid (Figure S4), overall, nuclei of EGFP^+^ cells seem smaller than the rest of the (GFP^−^) population in both genotypes (Figure S4B). We conclude that, in contrast to intestine, the number of Lgr5^+^ cells in *Ercc1^Δ^* ^/−^ liver is increased. A fraction of LSCs suffers from polyploidy most likely as a consequence of accumulated DNA damage suggesting limited functional potential.

### 2.4 Functional exhaustion of liver but not intestinal SC populations of progeroid mice

**FIGURE 4:**
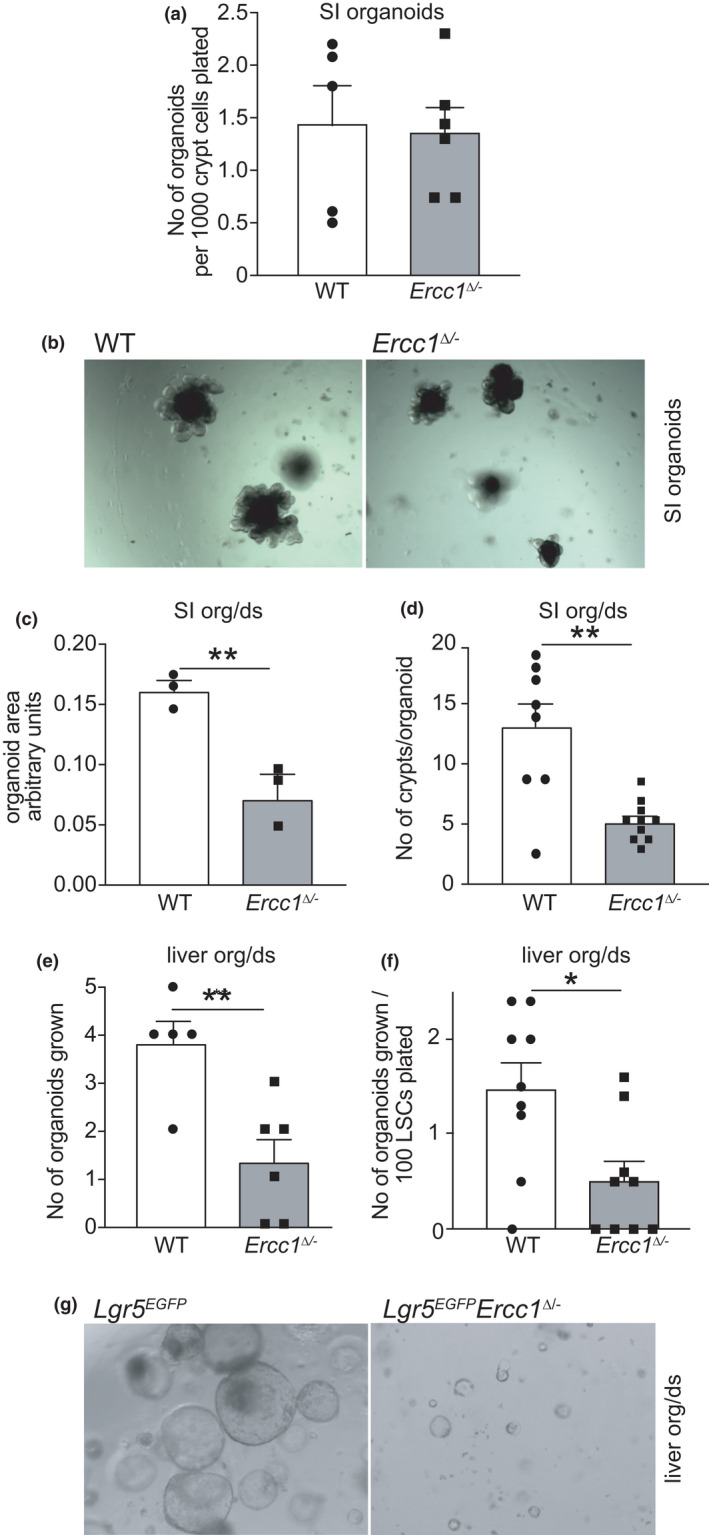
*Ex vivo* culture of intestinal and liver SCs from *Ercc1^Δ^* ^/−^ mice. (a) Organoids grown from intestinal crypt cell suspensions, derived from 15‐week‐old *Ercc1^Δ^* ^/−^ and wt mice, after 9 days in culture, *p* = not significant (*n* = 5 mice per genotype). (b) *In vitro* cultures of freshly isolated crypts from 15‐week‐old mice of the indicated genotypes. Images after 9 days in culture. (c) Average organoid size of the indicated genotypes after 9 days in culture (*n* = 3 independent cultures derived from different mice per group). (d) Average number of crypts budded in organoids of the indicated genotypes after 9 days in culture (at least two cultures for each mouse and 3 mice per genotype). (e) Number of organoids grown after plating bile cell containing liver cell suspensions, derived from 15‐week‐old *Ercc1^Δ^* ^/−^ mice and wt controls (at least two cultures for each mouse and 3 mice per genotype). Organoids were counted at Day 7 of culture. (f) Number of liver organoids grown in secondary cultures, after plating single liver stem cells derived from a primary organoid culture (at least three cultures were measured for each mouse and 3 mice per genotype). Organoids were counted at Day 7 of culture. (g) *In vitro* cultures of liver organoids grown from bile cell suspensions derived from livers of 15‐week‐old mice of the indicated genotypes. Images were taken after 7 days in culture. Data: mean ± SEM. *p < 0.05, **p < 0.01

To assess functional consequences of *Ercc1* deficiency on SCs, we examined the ability of mutant and WT ISCs to expand into organoids. After seeding an equivalent number of crypts, organoid‐forming capacity of ISCs seems similar for both genotypes (Figure 4a). However, mutant differentiated organoids, appeared smaller than WT with lower numbers of organoid‐budding crypts (Figure 4b–d). Culturing crypts in 3% O_2_ did not significantly improve *Ercc1^Δ^* ^/−^ (or WT) organoid development (Figure S5A) ruling out that it was caused by increased oxidative stress due to culturing in atmospheric oxygen, as previously noted in *Ercc1* ^−/−^ primary mouse embryonic fibroblasts (Fuhrmann‐Stroissnigg et al., 2017; Niedernhofer et al., 2006; Tilstra et al., 2012). Therefore, the reduced number of ICSs in isolated crypts in *Ercc1^∆^* ^/−^ intestine (Figure 3b,c) might explain the difference in organoid size.

To probe *Ercc1^Δ^* ^/−^ LSC self‐renewal potential, we used *ex vivo* organoid cultures (Huch et al., 2013). After seeding equal numbers of isolated liver cells, bile cells from mutant liver yielded far fewer organoids than controls (Figure 4e). To examine the possible effect of a difference in starting amount of bile cells (Figure 2f), from which organoids spawn, we plated secondary cultures from the organoids. This showed that organoid‐forming ability of *Ercc1^Δ^* ^/−^ LSCs is impaired (Figure 4f). Moreover, mutant organoids are dramatically smaller (Figure 4g). Both phenotypes were not rescued using low (3%) oxygen culture conditions (Figure S5B).

To conclude, whereas organoid‐forming capacity seems unaltered, *Ercc1^∆^* ^/−^ ISCs show reduced growth and formation of budding crypts. Mutant LSCs, however, display strongly diminished ability to form organoids, and drastically compromised development, suggesting SC exhaustion.

### 2.5 Diverse functional outcomes of DNA damage in *Ercc1^Δ^* ^/−^ intestinal and liver SCs

**FIGURE 5:**
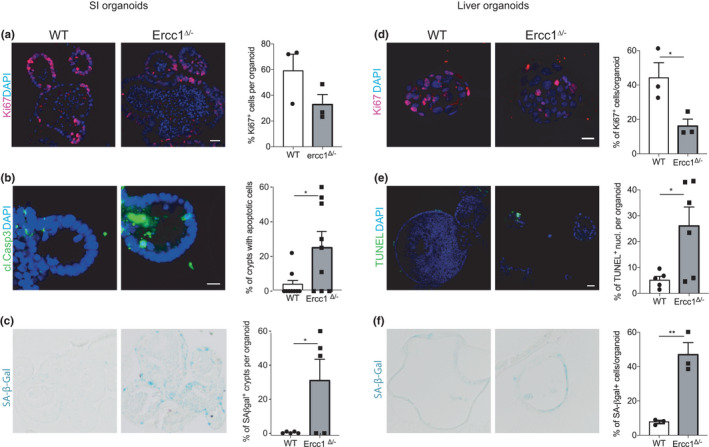
Cell fate phenotypes in *Ercc1^Δ^* ^/−^ small intestinal and liver organoids. (a) Proliferation index of *Ercc1^Δ^* ^/−^ intestinal organoids. Immunostaining for Ki67 (red) in *Ercc1^Δ^* ^/−^ and wt control SI organoids in medium without Wnt3a supplementation. Bar 10 μm. Right, quantitation of proliferative, Ki67^+^ cells in organoids of the indicated genotypes (*n* = 3 mice per genotype). (b) DNA damage responses in *Ercc1^Δ^* ^/−^ SI organoids. Images of crypts of organoids, derived from 15‐week‐old *Ercc1^Δ^* ^/−^ and control mouse intestines, immunostained for cleaved Caspase‐3. Bar 10 μm. SI organoid crypts containing TUNEL^+^ cells. Each dot represents the measurement of individual organoid cultures and data from a sample size of 3 mice per genotype. (c) Images of SI organoids from 15‐week‐old mutant and wt mice, stained for senescence‐associated β‐galactosidase (SA‐βGal) activity. Crypts positive for SA‐βGal in organoids of the indicated genotypes. Each dot individual culture of organoids, data from 3 mice per genotype. (d) Immunostaining for Ki67 of liver organoids from 15‐week‐old *Ercc1^Δ^* ^/−^ and wt mice. Bar 20μm. Proliferation index of organoids (*n* = 3 mice per genotype). (e) Apoptosis in *Ercc1^Δ^* ^/−^ and wt liver organoids from 15‐week‐old mice. Bar 50 μm. Percentage TUNEL^+^ cells per organoid (*n* = 5 mice per genotype). (f) Histochemical staining for SA‐β‐Gal activity. Percentage of SA‐β‐Gal^+^ cells per *Ercc1^Δ^* ^/−^ and wt liver organoid (*n* = 3 mice per genotype). Data: mean ± SEM. *p < 0.05, **p < 0.01

*Ercc1^Δ^* ^/−^ SI organoids show Ki67^+^ cell content similar to WT (Figure 5a), indicating that proliferative capacity *in vitro* is largely unaffected, consistent with the *in vivo* findings. TUNEL and cleaved Caspase‐3 staining revealed that *Ercc1^Δ^* ^/−^ SI organoid crypts contain cells undergoing apoptosis (Figure 5b), also recapitulating the mouse tissue phenotype. Interestingly, mutant crypts were found positive for senescence‐associated β‐galactosidase (SA‐β‐Gal), indicating the features of senescence (Figure 5c).

In contrast, *Ercc1^Δ^* ^/−^ liver organoids display considerably less Ki67^+^ cells (Figure 5d) and a significant fraction of SCs was apoptotic or positive for senescence (Figure 5e,f), the latter deviating from our findings in 15‐week‐old liver (Figure S3A–F), suggesting that culture conditions are more stressful than *in situ* and reach the threshold for senescence. LSCs show moderately compromised proliferation (as assessed by EdU incorporation Figure 5d and S6A,B) and γH2AX foci indicative of DNA breaks (Figure S6C). We found little overlap of γH2AX signal with replicating cells (Figure S6D), consistent with the notion that *Ercc1* defects also include repair systems such as TCR (amplified by GG‐NER deficiency) linked with transcription, affecting all cell cycle stages.

Flow cytometry for polyploidy showed a trend for more >4*n* cells in *Ercc1^Δ^* ^/−^ organoids, however, increased DNA content did not reach statistical significance (Figure S6E). Therefore, we measured the perimeter of EdU^+^ nuclei 48 hours after a short pulse, analysing exclusively cells that have undergone replication. Indeed, a significant fraction of *Ercc1^Δ^* ^/−^ LSC nuclei seems to be larger and some very large, indicating that they further progress in polyploidization *in vitro* (Figure S6F,G). Staining for albumin, a marker of differentiated hepatocytes, showed that *Ercc1^Δ^* ^/−^ LSCs in culture do not spontaneously undergo differentiation (data not shown). Collectively, we reveal diverse DNA damage responses in *Ercc1^Δ^* ^/−^ SI and liver SCs in organoid cultures, affecting stemness and regenerative capacity.

### 2.6 Comparison of functional DNA repair capacity of intestinal and liver stem cells

**FIGURE 6:**
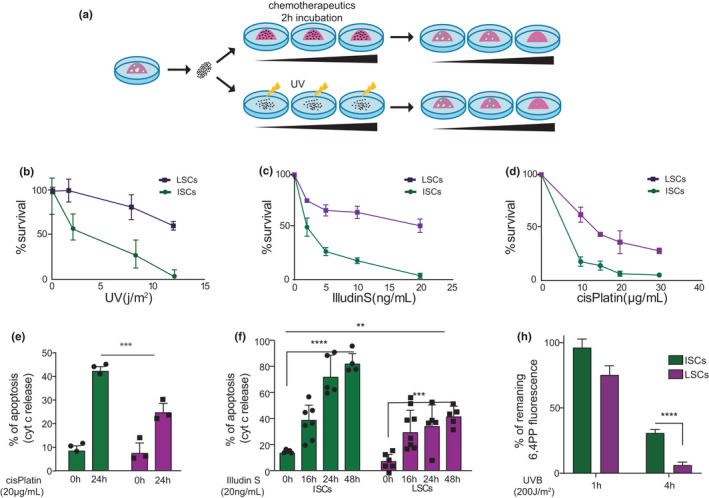
Differential responses of repair‐proficient liver and intestinal stem cells to exogenous damage. (a) Schematic of experimental set‐up for organoid formation assay following DNA damage induction. For each genotoxic agent, cultures from 2 wt mice were used and for each mouse, organoids grown in triplicates were quantified. (b‐d) Clonogenic survival of wt liver and intestinal stem cells upon UVC‐exposure (b); 2 h. treatment with the indicated concentrations of illudin S, which is only removed by transcription‐coupled repair (c) and the DNA‐crosslinking agent cisplatin (d). Organoids from single stem cell containing cultures were counted 5 days after treatment (*n* = 2 mice per group). (e, f) Number of stem cells that have lost cytochrome c at various time points following treatment with cisplatin (e) or illudin S (f). Data: means and SD of replicate samples from 2 mice in total. (g) Quantitation of 6,4‐photoproducts’ immunosignal in organoid stem cells at the indicated time points following UVB irradiation. Plotted is the percentage of remaining fluorescence relative to the 5 min time point from at least 500 nuclei of separate organoids of each mouse (3 mice per time point). p‐values were calculated using Student's *t* test. Data: mean ± SEM unless otherwise specified. **p < 0.01, ***p < 0.001, ****p < 0.0001

Since unrepaired DNA damage is the most logical culprit for the SC phenotypes of *Ercc1^Δ^* ^/−^ mice, we wished to examine SC responses to different classes of DNA‐damaging agents. We chose UV that causes base‐pair‐disrupting photoproducts mainly repaired by GG‐NER; Illudin S, which induces lesions that block transcription and are repaired by TCR (Jaspers et al., 2002); and interstrand cross‐links inflicted by cisplatin, which require cross‐link repair and mostly affect replication. *Ercc1* mutants are deficient in all of these pathways (Marteijn et al., 2014; Niedernhofer et al., 2006). We assessed the ability of cultured repair‐proficient ISCs and LSCs to survive and expand into organoids, following increasing doses of the above genotoxins. Surprisingly, although the intestine compared with liver shows less (premature) ageing in WT and when *Ercc1* is mutated, ISCs appeared consistently more sensitive to all genotoxins than LSCs (Figure 6a–d). ISCs were previously shown to be more sensitive to γ‐irradiation (Barker, 2014). Cytochrome C release following treatment with illudin S and cisplatin illustrates the hypersensitivity of ISCs to apoptotic cell death compared to LSCs (Figure 6e,f). Apparently, after equal DNA damage ISCs opt for cell death and LSCs prioritize survival. We wondered whether this differential genotoxin sensitivity correlates with differential repair capacity. Previously, we noted that core NER genes are higher expressed in LSCs than ISCs (Jager et al., 2019). However, expression levels do not always correlate with repair efficiency (Naipal et al., 2015). We examined 6,4‐photoproduct resolution after treatment of SCs with UVB, reflecting mainly GG‐NER and to a lesser extent TCR activities. As shown by the remaining 6,4‐photoproduct, immunosignals LSCs appear superior in repairing these lesions (Figure 6g). Inefficient repair likely enhances the propensity of ISCs to undergo apoptosis. Taken together, despite the virtual absence of accelerated ageing features in intestine, ISCs appear inferior in major DNA repair systems when assayed in parallel with LSCs.

## 3 DISCUSSION

At tissue level, we found no overt features of accelerated ageing in *Ercc1^Δ^* ^/−^ SI. Since *Ercc1^Δ^* ^/−^ mutants include cross‐link repair and GG‐NER defects, which are highly relevant for replication, this is unexpected as intestine is the most proliferative organ. Cell death affects many tissues of naturally aged mice (Pollack et al., 2002), including SI (Martin et al., 1998; Nalapareddy et al., 2017; Steegenga et al., 2017). Interestingly, *Ercc1^Δ^* ^/−^ crypts, transient amplifying zone and villi, reveal increased cell death in all stages of intestinal development, consistent with underlying stochastic damage events. Overall regenerative potential of the crypts is maintained, in line with the plasticity reported for ISC populations (Ritsma et al., 2014; Tetteh et al., 2016; Tian et al., 2011) and the presence of slow‐cycling, reserve ISCs (Barriga et al., 2017). Although we cannot exclude alterations in cell cycle rates, elevated spontaneous apoptosis had no detectable effect on tissue proliferation. Possibly, this went undetected amidst the high cell renewal in intestine. In sharp contrast, *Ercc1^Δ^* ^/−^ liver displays dramatic ageing pathology (Gregg et al., 2012; Vermeij, Dollé, et al., 2016; Weeda et al., 1997). Here, we show that apoptosis is prominent in nearly all liver cell types and p21 elevated in a fraction of cells. The intercellular heterogeneity in p21 levels parallels p53 in *Ercc1* ^−/−^ liver (McWhir et al., 1993), consistent with stochastic DNA damage. The p21 response likely reflects resistance to apoptosis—explaining the modest increase in hepatocyte death, prior to reaching moribund stages, where senescence and apoptosis are strongly raised (Gregg et al., 2012; Yousefzadeh et al., 2021). The elevated proliferative index, persistent damage and different DDR, likely explain the increased mutations in LSCs compared with ISCs (Jager et al., 2019).

At the SC level, *Ercc1^Δ^* ^/−^ ISCs are diminished in number, possibly through apoptosis. However, remaining ISCs appear functionally normal. Nevertheless, the number of quiescent *Lgr5* ^+^ cells is reduced in *Ercc1^Δ^* ^/−^ crypts, which can be either due to apoptosis and/or cell cycle entry to restore regenerative capacity. In contrast, *Ercc1^Δ^* ^/−^ liver displays increased levels of *Lgr5* ^+^ cells, but these cells show evidence of impaired functionality: some are polyploid—indicative of genomic stress. The bile cell population appears compromised. Elevated apoptosis and p21 upregulation point to loss of stemness properties and reduced regenerative potential.

At the organoid level, *Ercc1^Δ^* ^/−^ ISCs show ~WT ability to form organoids, which are smaller and contain less crypts, suggesting reduced ability to grow and differentiate. Although proliferative potential is marginally decreased, apoptosis and senescence in these crypts are elevated, in line with time‐dependent stochastic DNA damage causing the organoid's limited net growth under culture conditions. In comparison, LSCs appear to be functionally even more compromised in all regards: they form fewer organoids, which are also much smaller and contain fewer proliferative cells, suggesting proliferative exhaustion. Apoptosis, senescence, polyploidy and irregularly shaped nuclei are also increased in liver organoids, accounting for their poor growth. Increased γH2AX foci, mainly in non‐replicating cells provides evidence for DNA damage likely related to transcription (rather replication) stress, a genome‐wide ageing phenomenon, first discovered in *Ercc1^Δ^* ^/−^ and *Xpg* ^−/−^ liver and subsequently also in natural ageing (Vermeij, Dollé, et al., 2016), which is associated with functional decline, cell cycle arrest, senescence and cell death. This novel ageing feature occurs primarily in organs with low cell renewal (since DNA replication dilutes damage) and affects expression preferentially of large genes, consistent with accumulating random DNA lesions compromising transcription in a gene‐length‐dependent manner (Vermeij, Dollé, et al., 2016). The origin of the endogenous DNA damage is unclear, but in our *in vitro* studies appears largely independent from O_2_ levels (see Figure S5b), fitting our observation (Milanese et al., 2019) that TCR‐ defects trigger a potent anti‐oxidant defence (Garinis et al., 2009; Niedernhofer et al., 2006; van der Pluijm et al., 2007).

Directly comparing the DDR and repair properties of WT ISCs and LSCs, we show that ISCs are more sensitive to diverse genotoxins attesting different repair systems. Individual ISC types have distinct damage sensitivities (Shivdasani, 2014). Quiescent *Lgr5* ^+^ cells appear more resistant, while fast‐dividing ISCs have a low apoptotic threshold, as shown for ionizing radiation (Barker, 2014; Barriga et al., 2017). Consistent with low expression of NER genes (Jager et al., 2019), we find ISCs to be less efficient in repair of UV‐lesions and more sensitive to UV, illudin S and cisplatint damages eliminated by GG‐NER, TCR and cross‐link repair, respectively, supporting the idea that the entire repair machinery is functionally inferior in ISCs.

As liver exhibits much more ageing features than intestine, one might expect genome maintenance in ISCs to be superior. However, in a direct comparison, repair in LSCs appears superior. This explains why *Ercc1* (and all combined TCR/GG‐NER) mutants display much less ageing in intestine than liver (and, as evident from human and mouse TCR/GG‐NER mutants, also e.g., neurons and kidney). Apparently, organs utilize different anti‐ageing strategies, involving DDR mechanisms. This raises the question—why do ISCs not invest maximally in DNA repair? One reason may be their tight replicative and differentiation schedule imposed by the rapid renewal of intestinal epithelium, which may not permit spending much time and energy for repair. Intestinal epithelial cells have only a lifespan of only 3–5 days (Gehart & Clevers, 2015) with limited time for DNA damage to accumulate, rendering repair less critical, and hence, prefer they opt for apoptosis. The high proliferation rate of ISCs and the reserve capacity probably can easily compensate for the loss of a relatively small fraction of cells.

The above strategy may be less suitable when cell turnover is slower with more time for lesions to accrue, increasing dependence on repair. This matches with the severe functional and numerical SC exhaustion in *Ercc1* mutant mice for the hematopoietic system (Cho et al., 2013; Prasher et al., 2005; Rossi et al., 2007), in which SCs have an estimated average turn‐over of ~2 months. Presumably, exhaustion of HSCs in the *Ercc1* mutant is largely caused by its replication‐associated cross‐link repair defect, as in Fanconi's anaemia (FA), in line with the notion that defects in the ERCC1 partner XPF can cause FA (Bogliolo et al., 2013; Kuraoka et al., 2000).

Presumably, the investments in high intestinal tissue turn‐over cannot be afforded by many other organs and cell turn‐over is incompatible with the primary function of, for example most post‐mitotic neurons. Moreover, the intestine resides in a very hostile environment, including the microbiome, with all metabolites passing through its epithelium rendering the organ with the highest exposure to exogenous compounds, explaining its 3–5 day cell turn‐over. Although liver, as main detoxification organ, is also exposed to numerous toxics, we assume that its superior repair and high damage tolerance enable hepatocytes to live longer. Since cells in liver replicate only occasionally, they must rely on replication‐independent DNA repair pathways. Particularly, their transcribed compartment is vital for sustained functionality, explaining why TCR mutants display a segmental progeroid phenotype strongly biased towards post‐mitotic organs and tissues, such as the neuronal system, liver, kidney, fat tissue and skeleton, but less bone marrow and intestine (Lans et al., 2019). A combined TCR/GG‐NER deficiency as in *Ercc1* mutants dramatically augments these segmental ageing features, as all GG‐NER lesions also hamper transcription (Andressoo et al., 2006; de Boer et al., 2002).

### 3.1 A model for the relationship between DNA damage responses and tissue‐specific ageing trajectories

**FIGURE 7:**
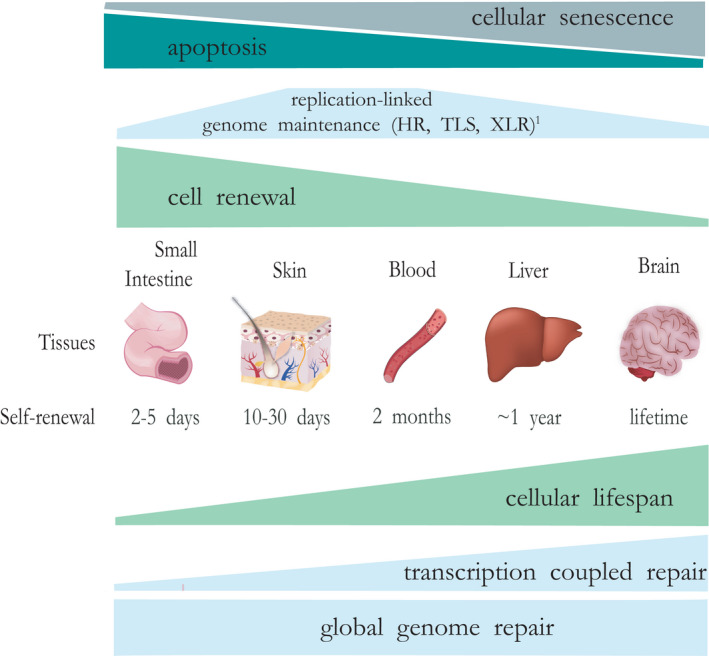
Tentative model for organ‐specific anti‐ageing strategies. Remaining cellular lifespan largely determines which DNA damage response strategy is preferred by organs/tissues to counteract ageing. For intestine, cell death is preferred, as cells have to function only for 3–5 days and cell loss can be compensated by increased (stem) cell proliferation. Obviously, this is very energy‐demanding (daily a human body produces 200 gram intestinal epithelium) and unaffordable for many organs. Other tissues with continuous but slower cell renewal such as the hematopoietic system (average cell turn‐over ~2 months) rely mostly on replication‐related repair (such as NHEJ/HR and XLR) and apoptosis (Hoeijmakers, 2001). Skin, as an organ with high UV exposure and also intermediate cell renewal combines GG‐NER and TCR with apoptosis and premature differentiation of damaged stem cells (Kim et al., 2020). Finally, tissues with slow (e.g., liver, on average ~1 year) or no cell turn‐over (e.g., the central nervous system, life time) depend on constitutive (cell cycle independent) DNA repair systems, most notably TCR to permit long‐term unperturbed use of the transcribed compartment of the genome, needed for sustained proper cellular functioning. Global genome repair systems (base and nucleotide excision repair) are probably important for all organs and tissues for preventing mutagenesis and permit survival. TLS allows replication bypass of lesions to rescue stalled replication and cellular proliferative capacity, however, at the expense of elevated mutagenesis and cancer risk. Cellular (replicative) senescence opposes cell death in most organs. This model explains the segmental nature of repair‐deficient progeroid syndromes, in which inherited deficiencies in different repair systems are associated with a different subset of organs and tissues displaying accelerated ageing. ^1^Not including mismatch repair, which is a replication error correction system, important for preventing mutations and cancer, particularly in highly proliferative tissues such as intestine. GG‐NER, global genome nucleotide excision repair; NHEJ/HR, Non‐Homologous End‐Joining/Homologous recombination repair two pathways for double strand break repair; TCR, transcription‐coupled repair; TLS, translesion synthesis; XLR, cross‐link repair

Based on the central role of DNA damage for ageing, we propose a tentative model (Figure 7), in which different DNA repair and damage response systems linked with replication and transcription (cell cycle arrest, senescence, cell death and mutagenesis) are differentially employed as anti‐ageing/cancer strategies. Very frequent replicating cells with a short lifespan, as in intestinal epithelium opt primarily for apoptosis and cell replacement. On the other extreme, liver (and by inference from the phenotypes of mouse and human progeroid mutants with defects in the same repair pathways also neurons, kidney, and skeleton with mostly post‐mitotic cells with a long lifespan invest in cell survival by damage sensing and repair primarily linked with transcription and the transcribed compartment (i.e., the most important functional part), involving TCR with the help of global genome repair systems (GG‐NER and base excision repair). They also may have high damage tolerance and consequent senescence levels. Tissues with an intermediate stem cell turn‐over and lifespan, such as the hematopoietic system, invoke both apoptosis and replication‐linked DDR such as cross‐link and double‐strand break repair by non‐homologous‐endjoing and homologous recombination repair. Global genome and replication‐linked repair and response mechanisms are critical for preventing mutagenesis and cancer. Such a model provides an explanation for the segmental ageing phenotypes of progeroid syndromes in man and mouse mutants in which deficiencies in different DNA repair systems are associated with a different subset of accelerated ageing symptoms. Natural variation in genome maintenance mechanisms, metabolism and exogenous exposure likely contributes to the different ageing trajectories between organs and inter‐individual differences in normal ageing. Finally, we show that organoid cultures from progeroid mice faithfully recapitulate various ageing features and may be useful tools for studying regenerative interventions.

## 4 METHODS

### 4.1 Mice

All animals were housed in the Erasmus Medical experimental animal center and handled in accordance with the recommendations and regulations of the animal ethical committee (DEC‐consult) and national/EU legislation. *Ercc1^∆^* ^/−^ mice were bred in a hybrid F1 genetic background (FVB/N:C57Bl/6J) and genotyped as previously described (Vermeij, Dollé, et al., 2016). The *Lgr5^EGFP^* ^−^ *^IRES^* ^−^ *^CRE2^* transgenic mouse line has been previously described (Barker et al., 2007). *Lgr5^EGFP^* ^−^ *^IRES^* ^−^ *^CRE2^ Ercc1^Δ^* ^/−^ mice were generated by first crossing male *Ercc1* ^+/−^ mice with female *Lgr5^EGFP^* ^−^ *^IRES^* ^−^ *^CRE2^*, all in a pure C57BL/6J background. Female *Lgr5^EGFP^* ^−^ *^IRES^* ^−^ *^CRE2^ Ercc1* ^+/−^ C57BL/6J progeny were subsequently crossed with male *Ercc1^∆^* ^/+^ mice, in a pure FVB background and the desired *Lgr5^EGFP^* ^−^ *^IRES^* ^−^ *^CRE2^ Ercc1^Δ^* ^/−^ offspring were obtained in a 50/50 F1 C57BL6J/FVB hybrid background. *Ercc1^∆^* ^/+^ and *Ercc1* ^+/−^ mice have been previously described (Weeda et al., 1997).

### 4.2 Immunohistochemistry and immunocytochemistry, microspcopy, FACS and flow cytometric analysis

Immunohistochemistry and immunocytochemistry of intestinal and liver tissues were done according to established procedures and detailed in supplementary materials, including the specific reagents (e.g., antibodies) used. Stainings of paraffin‐embedded SI and liver tissue sections and liver organoids for senescence markers were performed as described (Baar et al., 2017). Procedures for microscopy, FACS and flow cytometric analysis and equipment are specified in Supplementary materials.

### 4.3 Apoptosis

TUNEL staining of tissue sections and organoid cultures was performed using an *in situ* cell death kit (Roche) or the Apoptag Plus Peroxidase *in situ* apoptosis detection kit (Millipore). Apoptosis of ISCs and LSCs after treatment with illudin S and cisplatin was assessed by staining for cytochrome c of single ISC and LSC suspensions—derived from undifferentiated organoid cultures—embedded in Matrigel using standard immunofluorescence protocol for cells (Baar et al., 2017).

### 4.4 *In* *vitro* small intestinal organoid culture

Intestinal organoid formation from crypts was performed as described (Sato et al., 2009). A total of 1000 crypts was mixed with 50 μl of Matrigel and plated in 24‐well plates. Intestinal organoids were grown in ENR medium (Advanced DMEM/F12, B27 and N2 supplement (Invitrogen), 50 ng μl^−1^ epidermal growth factor, 100 ng ml^−1^ Noggin (Peprotech), 500 ng ml^−1^ R‐spondin‐1, 1.25 mM *N*‐acetylcystein) supplemented with Wnt3a‐conditioned medium34 when appropriate to keep organoids in undifferentiated state. For organoid growth from single cell crypt suspensions, crypts were incubated with TrypLE (Invitrogen) and passed through a 40 μm cell strainer. Resulting cell suspensions were mixed with Matrigel and plated as described with supplementation of growth medium with 10μM Rock inhibitor Y27632 (Sigma). The medium was refreshed every two days. Organoids were passaged every 10 days.

### 4.5 *In* *vitro* liver organoid culture

Growth of organoid cultures from liver bile duct enriched cell suspensions was performed as described (Broutier et al., 2016; Huch et al., 2013, 2015). A total of 100,000 cells was mixed with 50 μl of Matrigel and plated on 24‐well plates. Culture medium was refreshed every two days, and organoids were passaged every 8 to 10 days. Growth of liver organoids from single cells was performed with fluorescence‐activated cell sorting of cell suspensions derived from TrypLE‐digested primary cultures.

### 4.6 EdU incorporation assay

In intestinal and liver organoid culture medium, EdU was added for 2 h at a final concentration of 10 μM, cultures were washed with PBS, replenished and at the appropriate time points, fixed with 4% paraformaldehyde in PBS for 15 min at room temperature. Edu immunofluorescence was performed using Click‐iT^®^ EdU Alexa Fluor^®^ Imaging Kit (Invitrogen), according to manufacturers’ instructions.

### 4.7 Genotoxic sensitivity assay

Organoids were dissociated into single cell suspension using TrypLE reagent and passed through a 40 μm cell strainer. Following cell counting, 10,000 single ISCs and 500 LSCs were resuspended in 20 μl Matrigel and plated in a 48‐well plate. Cisplatin and illudin S were diluted in HBSS at appropriate concentrations and added to each well. Two hours after treatment medium was removed and replaced with appropriate stem cell culture medium supplemented with 10μM Rock inhibitor Y27632 (Sigma). For ISC cultures, Gsk3 inhibitor CHIR‐9921 (Axon MedChem) was added to the culture medium at a final concentration of 10 μM.

For UV sensitivity assessment, suspensions of single stem cells were plated in 12‐well plates at different densities and centrifuged at 600 relative centrifugal force for 20 min. at 32°C, medium was carefully removed, and the cells at the well bottom were exposed to the indicated doses of UV (254nm, TUV Lamp Philips). Cells were collected in culture medium, centrifuged at 1500 rpm, resuspended in Matrigel and plated in triplicates in 48‐well plates. Organoids were counted 5 days after plating.

### 4.8 Assessment of DNA lesion resolution in SCs

Organoids were grown in 8‐well tissue culture slides, exposed to UVB (200J/m^2^, Philips 40W/12RS UVB lamp) and at selected time points fixed (2% formalin, 20 min. at RT), washed with PBS and 0.1 M glycine, permeabilized with 0,5% Triton‐X‐100 in PBS for 20 min. RT and treated with 2N HCL for 30 min to denature DNA. After extensive washes with PBS and incubation with blocking solution (1% BSA in PBS), 150μl of anti‐6,4‐PP antibody (COSMO‐BIO, Cat#CAC‐NM‐DND‐002) mix (1:500 in blocking solution) was added to each well, and the slides were transferred to a 37^0^C incubator for 3 h. Organoids were washed 3 times with PBST (PBS and 0.05% Triton‐X‐100) and incubated with secondary antibody (Alexa Fluor 555) diluted 1:100 in PBS for 2 h at 37°C. Nuclei were counterstained with Hoechst for 30min. Subsequently, Prolong Diamond mounting medium (Thermo Fisher) was added and slides were incubated overnight in a freezer. Images were captured with a confocal microscope. Fluorescence measurements and analysis were performed using image J software.

### 4.9 Statistics

Experimental sample size was not strictly chosen based on utilization of statistical methods prior to initiation of the study. Experiments were not standardly performed and analysed in a randomized, blinded fashion. Statistical analysis was performed with GraphPad Prism software. Student's *t* test and nonparametric Mann‐Whitney test were used to calculate *P* values. For multiple comparisons, two‐way ANOVA was implemented for evaluation of statistical significance (GraphPad PRISM).

For all other procedures, see Supplementary Materials.

## CONFLICT OF INTEREST

The authors declare no conflict of interest.

## AUTHOR CONTRIBUTIONS

M.V. designed experimental work, performed experiments, analysed data and wrote the manuscript. J.D. designed, performed and analysed experiments. M.B. performed senescence stainings. S.B. performed flow cytometry, A.M. Ki67, and GFP immunostainings and critically commented on experiments. E.K., R.v.B. and M.J. grown organoid cultures and provided expertise, reagents and protocols. S.M. commented on experiments, data analysis and participated in cell viability experiments. R.M.C.B. helped with the mouse experiments. W.P.V. provided data on p21 and apoptosis and commented on study design and manuscript. J.K. performed immunostainings. R.v.B. and E.C. were involved in the conceptual design. J.P. and J.H.J.H. conceived and supervised the study and wrote the manuscript.

## Supporting information

Fig S1

Fig S2

Fig S3

Fig S4

Fig S5

Fig S6

Supplementary Material

## Data Availability

The data that support the findings of this study are available from the corresponding author upon reasonable request.

## References

[acel13562-bib-0001] Ahmad, A. , Robinson, A. R. , Duensing, A. , van Drunen, E. , Beverloo, H. B. , Weisberg, D. B. , Hasty, P. , Hoeijmakers, J. H. , & Niedernhofer, L. J. (2008). ERCC1‐XPF endonuclease facilitates DNA double‐strand break repair. Molecular and Cellular Biology, 28, 5082–5092. 10.1128/MCB.00293-08 18541667 PMC2519706

[acel13562-bib-0002] Alyodawi, K. , Vermeij, W. P. , Omairi, S. , Kretz, O. , Hopkinson, M. , Solagna, F. , Joch, B. , Brandt, R. M. C. , Barnhoorn, S. , Vliet, N. , Ridwan, Y. , Essers, J. , Mitchell, R. , Morash, T. , Pasternack, A. , Ritvos, O. , Matsakas, A. , Collins‐Hooper, H. , Huber, T. B. , … Patel, K. (2019). Compression of morbidity in a progeroid mouse model through the attenuation of myostatin/activin signalling. Journal of Cachexia, Sarcopenia and Muscle, 10, 662–686. 10.1002/jcsm.12404 PMC6596402 30916493

[acel13562-bib-0003] Andressoo, J.‐O. , Mitchell, J. R. , de Wit, J. , Hoogstraten, D. , Volker, M. , Toussaint, W. , Speksnijder, E. , Beems, R. B. , van Steeg, H. , Jans, J. , de Zeeuw, C. I. , Jaspers, N. G. J. , Raams, A. , Lehmann, A. R. , Vermeulen, W. , Hoeijmakers, J. H. J. , & van der Horst, G. T. J. (2006). An Xpd mouse model for the combined xeroderma pigmentosum/Cockayne syndrome exhibiting both cancer predisposition and segmental progeria. Cancer Cell, 10, 121–132. 10.1016/j.ccr.2006.05.027 16904611

[acel13562-bib-0004] Baar, M. P. , Brandt, R. M. C. , Putavet, D. A. , Klein, J. D. D. , Derks, K. W. J. , Bourgeois, B. R. M. , Stryeck, S. , Rijksen, Y. , van Willigenburg, H. , Feijtel, D. A. , van der Pluijm, I. , Essers, J. , van Cappellen, W. A. , van IJcken, W. F. , Houtsmuller, A. B. , Pothof, J. , de Bruin, R. W. F. , Madl, T. , Hoeijmakers, J. H. J. , … de Keizer, P. L. J. (2017). Targeted apoptosis of senescent cells restores tissue homeostasis in response to chemotoxicity and aging. Cell, 169(132–147), e116. 10.1016/j.cell.2017.02.031 PMC5556182 28340339

[acel13562-bib-0005] Barker, N. (2014). Adult intestinal stem cells: critical drivers of epithelial homeostasis and regeneration. Nature Reviews Molecular Cell Biology, 15, 19–33. 10.1038/nrm3721 24326621

[acel13562-bib-0006] Barker, N. , van Es, J. H. , Kuipers, J. , Kujala, P. , van den Born, M. , Cozijnsen, M. , Haegebarth, A. , Korving, J. , Begthel, H. , Peters, P. J. , & Clevers, H. (2007). Identification of stem cells in small intestine and colon by marker gene Lgr5. Nature, 449, 1003–1007. 10.1038/nature06196 17934449

[acel13562-bib-0007] Barnhoorn, S. , Uittenboogaard, L. M. , Jaarsma, D. , Vermeij, W. P. , Tresini, M. , Weymaere, M. , Menoni, H. , Brandt, R. M. C. , de Waard, M. C. , Botter, S. M. , Sarker, A. H. , Jaspers, N. G. J. , van der Horst, G. T. J. , Cooper, P. K. , Hoeijmakers, J. H. J. , & van der Pluijm, I. (2014). Cell‐autonomous progeroid changes in conditional mouse models for repair endonuclease XPG deficiency. PLoS Genetics, 10, e1004686. 10.1371/journal.pgen.1004686 25299392 PMC4191938

[acel13562-bib-0008] Barriga, F. M. , Montagni, E. , Mana, M. , Mendez‐Lago, M. , Hernando‐Momblona, X. , Sevillano, M. , Guillaumet‐Adkins, A. , Rodriguez‐Esteban, G. , Buczacki, S. J. A. , Gut, M. , Heyn, H. , Winton, D. J. , Yilmaz, O. H. , Attolini, C.‐O. , Gut, I. , & Batlle, E. (2017). Mex3a marks a slowly dividing subpopulation of Lgr5+ intestinal stem cells. Cell Stem Cell, 20, 801–816.e7. 10.1016/j.stem.2017.02.007 28285904 PMC5774992

[acel13562-bib-0009] Beerman, I. , Seita, J. , Inlay, M. A. , Weissman, I. L. , & Rossi, D. J. (2014). Quiescent hematopoietic stem cells accumulate DNA damage during aging that is repaired upon entry into cell cycle. Cell Stem Cell, 15, 37–50. 10.1016/j.stem.2014.04.016 24813857 PMC4082747

[acel13562-bib-0010] Begus‐Nahrmann, Y. , Lechel, A. , Obenauf, A. C. , Nalapareddy, K. , Peit, E. , Hoffmann, E. , Schlaudraff, F. , Liss, B. , Schirmacher, P. , Kestler, H. , Danenberg, E. , Barker, N. , Clevers, H. , Speicher, M. R. , & Rudolph, K. L. (2009). p53 deletion impairs clearance of chromosomal‐instable stem cells in aging telomere‐dysfunctional mice. Nature Genetics, 41, 1138–1143. 10.1038/ng.426 19718028

[acel13562-bib-0011] Bogliolo, M. , Schuster, B. , Stoepker, C. , Derkunt, B. , Su, Y. , Raams, A. , Trujillo, J. P. , Minguillón, J. , Ramírez, M. J. , Pujol, R. , Casado, J. A. , Baños, R. , Rio, P. , Knies, K. , Zúñiga, S. , Benítez, J. , Bueren, J. A. , Jaspers, N. G. J. , Schärer, O. D. , … Surrallés, J. (2013). Mutations in ERCC4, encoding the DNA‐repair endonuclease XPF, cause Fanconi anemia. American Journal of Human Genetics, 92, 800–806. 10.1016/j.ajhg.2013.04.002 23623386 PMC3644630

[acel13562-bib-0012] Broutier, L. , Andersson‐Rolf, A. , Hindley, C. J. , Boj, S. F. , Clevers, H. , Koo, B. K. , & Huch, M. (2016). Culture and establishment of self‐renewing human and mouse adult liver and pancreas 3D organoids and their genetic manipulation. Nature Protocols, 11, 1724–1743. 10.1038/nprot.2016.097 27560176

[acel13562-bib-0013] Cho, J. S. , Kook, S. H. , Robinson, A. R. , Niedernhofer, L. J. , & Lee, B. C. (2013). Cell autonomous and nonautonomous mechanisms drive hematopoietic stem/progenitor cell loss in the absence of DNA repair. Stem Cells, 31, 511–525. 10.1002/stem.1261 23097336 PMC3582850

[acel13562-bib-0014] de Boer, J. , Andressoo, J. O. , de Wit, J. , Huijmans, J. , Beems, R. B. , van Steeg, H. , Weeda, G. , van der Horst, G. T. J. , van Leeuwen, W. , Themmen, A. P. N. , Meradji, M. , & Hoeijmakers, J. H. J. (2002). Premature aging in mice deficient in DNA repair and transcription. Science, 296, 1276–1279. 10.1126/science.1070174 11950998

[acel13562-bib-0015] de Laat, W. L. , Jaspers, N. G. , & Hoeijmakers, J. H. (1999). Molecular mechanism of nucleotide excision repair. Genes & Development, 13, 768–785. 10.1101/gad.13.7.768 10197977

[acel13562-bib-0016] Dollé, M. E. T. , Kuiper, R. V. , Roodbergen, M. , Robinson, J. , de Vlugt, S. , Wijnhoven, S. W. P. , Beems, R. B. , de la Fonteyne, L. , de With, P. , van der Pluijm, I. , Niedernhofer, L. J. , Hasty, P. , Vijg, J. , Hoeijmakers, J. H. J. , & van Steeg, H. (2011). Broad segmental progeroid changes in short‐lived *Ercc1 ^−/Δ7^ * mice. Pathobiology of Aging & Age‐related Diseases, 1(1), 7219. 10.3402/pba.v1i0.7219 PMC3417667 22953029

[acel13562-bib-0018] Flach, J. , Bakker, S. T. , Mohrin, M. , Conroy, P. C. , Pietras, E. M. , Reynaud, D. , Alvarez, S. , Diolaiti, M. E. , Ugarte, F. , Forsberg, E. C. , Le Beau, M. M. , Stohr, B. A. , Méndez, J. , Morrison, C. G. , & Passegué, E. (2014). Replication stress is a potent driver of functional decline in ageing haematopoietic stem cells. Nature, 512, 198–202. 10.1038/nature13619 25079315 PMC4456040

[acel13562-bib-0019] Fuhrmann‐Stroissnigg, H. , Ling, Y. Y. , Zhao, J. , McGowan, S. J. , Zhu, Y. I. , Brooks, R. W. , Grassi, D. , Gregg, S. Q. , Stripay, J. L. , Dorronsoro, A. , Corbo, L. , Tang, P. , Bukata, C. , Ring, N. , Giacca, M. , Li, X. , Tchkonia, T. , Kirkland, J. L. , Niedernhofer, L. J. , & Robbins, P. D. (2017). Identification of HSP90 inhibitors as a novel class of senolytics. Nature Communications, 8, 422. 10.1038/s41467-017-00314-z PMC5583353 28871086

[acel13562-bib-0020] Garinis, G. A. , Uittenboogaard, L. M. , Stachelscheid, H. , Fousteri, M. , van Ijcken, W. , Breit, T. M. , van Steeg, H. , Mullenders, L. H. F. , van der Horst, G. T. J. , Brüning, J. C. , Niessen, C. M. , Hoeijmakers, J. H. J. , & Schumacher, B. (2009). Persistent transcription‐blocking DNA lesions trigger somatic growth attenuation associated with longevity. Nature Cell Biology, 11, 604–615. 10.1038/ncb1866 19363488 PMC2782455

[acel13562-bib-0021] Gehart, H. , & Clevers, H. (2015). Repairing organs: lessons from intestine and liver. Trends in Genetics, 31, 344–351. 10.1016/j.tig.2015.04.005 25989898

[acel13562-bib-0023] Gilgenkrantz, H. , & Collin de l'Hortet, A. (2018). Understanding Liver Regeneration: From Mechanisms to Regenerative Medicine. The American Journal of Pathology, 188, 1316–1327. 10.1016/j.ajpath.2018.03.008 29673755

[acel13562-bib-0024] Gillet, L. C. , & Scharer, O. D. (2006). Molecular mechanisms of mammalian global genome nucleotide excision repair. Chemical Reviews, 106, 253–276. 10.1021/cr040483f 16464005

[acel13562-bib-0025] Gregg, S. Q. , Gutiérrez, V. , Rasile Robinson, A. , Woodell, T. , Nakao, A. , Ross, M. A. , Michalopoulos, G. K. , Rigatti, L. , Rothermel, C. E. , Kamileri, I. , Garinis, G. A. , Beer Stolz, D. , & Niedernhofer, L. J. (2012). A mouse model of accelerated liver aging caused by a defect in DNA repair. Hepatology, 55, 609–621. 10.1002/hep.24713 21953681 PMC3250572

[acel13562-bib-0026] Hoeijmakers, J. H. (2001). Genome maintenance mechanisms for preventing cancer. Nature, 411, 366–374. 10.1038/35077232 11357144

[acel13562-bib-0027] Hoeijmakers, J. H. (2009). DNA damage, aging, and cancer. The New England Journal of Medicine, 361, 1475–1485. 10.1056/NEJMra0804615 19812404

[acel13562-bib-0028] Huch, M. , Dorrell, C. , Boj, S. F. , van Es, J. H. , Li, V. S. W. , van de Wetering, M. , Sato, T. , Hamer, K. , Sasaki, N. , Finegold, M. J. , Haft, A. , Vries, R. G. , Grompe, M. , & Clevers, H. (2013). In vitro expansion of single Lgr5+ liver stem cells induced by Wnt‐driven regeneration. Nature, 494, 247–250. 10.1038/nature11826 23354049 PMC3634804

[acel13562-bib-0029] Huch, M. , Gehart, H. , van Boxtel, R. , Hamer, K. , Blokzijl, F. , Verstegen, M. M. A. , Ellis, E. , van Wenum, M. , Fuchs, S. A. , de Ligt, J. , van de Wetering, M. , Sasaki, N. , Boers, S. J. , Kemperman, H. , de Jonge, J. , Ijzermans, J. N. M. , Nieuwenhuis, E. E. S. , Hoekstra, R. , Strom, S. , … Clevers, H. (2015). Long‐term culture of genome‐stable bipotent stem cells from adult human liver. Cell, 160, 299–312. 10.1016/j.cell.2014.11.050 25533785 PMC4313365

[acel13562-bib-0030] Jager, M. , Blokzijl, F. , Kuijk, E. , Bertl, J. , Vougioukalaki, M. , Janssen, R. , Besselink, N. , Boymans, S. , de Ligt, J. , Pedersen, J. S. , Hoeijmakers, J. , Pothof, J. , van Boxtel, R. , & Cuppen, E. (2019). Deficiency of nucleotide excision repair is associated with mutational signature observed in cancer. Genome Research, 29, 1067–1077. 10.1101/gr.246223.118 31221724 PMC6633256

[acel13562-bib-0031] Jaspers, N. G. , Raams, A. , Kelner, M. J. , Ng, J. M. , Yamashita, Y. M. , Takeda, S. , McMorris, T. C. , & Hoeijmakers, J. H. (2002). Anti‐tumour compounds illudin S and Irofulven induce DNA lesions ignored by global repair and exclusively processed by transcription‐ and replication‐coupled repair pathways. DNA Repair, 1, 1027–1038. 10.1016/S1568-7864(02)00166-0 12531012

[acel13562-bib-0032] Kim, D. E. , Dollé, M. E. T. , Vermeij, W. P. , Gyenis, A. , Vogel, K. , Hoeijmakers, J. H. J. , Wiley, C. D. , Davalos, A. R. , Hasty, P. , Desprez, P.‐Y. , & Campisi, J. (2020). Deficiency in the DNA repair protein ERCC1 triggers a link between senescence and apoptosis in human fibroblasts and mouse skin. Aging Cell, 19, e13072. 10.1111/acel.13072 31737985 PMC7059167

[acel13562-bib-0033] Kuraoka, I. , Kobertz, W. R. , Ariza, R. R. , Biggerstaff, M. , Essigmann, J. M. , & Wood, R. D. (2000). Repair of an interstrand DNA cross‐link initiated by ERCC1‐XPF repair/recombination nuclease. The Journal of Biological Chemistry, 275, 26632–26636. 10.1074/jbc.C000337200 10882712

[acel13562-bib-0034] Lans, H. , Hoeijmakers, J. H. J. , Vermeulen, W. , & Marteijn, J. A. (2019). The DNA damage response to transcription stress. Nature Reviews Molecular Cell Biology, 20(12), 766–784. 10.1038/s41580-019-0169-4 31558824

[acel13562-bib-0035] Lavasani, M. , Robinson, A. R. , Lu, A. , Song, M. , Feduska, J. M. , Ahani, B. , Tilstra, J. S. , Feldman, C. H. , Robbins, P. D. , Niedernhofer, L. J. , & Huard, J. (2012). Muscle‐derived stem/progenitor cell dysfunction limits healthspan and lifespan in a murine progeria model. Nature Communications, 3, 608. 10.1038/ncomms1611 PMC3272577 22215083

[acel13562-bib-0036] Marteijn, J. A. , Lans, H. , Vermeulen, W. , & Hoeijmakers, J. H. (2014). Understanding nucleotide excision repair and its roles in cancer and ageing. Nature Reviews Molecular Cell Biology, 15, 465–481. 10.1038/nrm3822 24954209

[acel13562-bib-0037] Martin, K. , Kirkwood, T. B. , & Potten, C. S. (1998). Age changes in stem cells of murine small intestinal crypts. Experimental Cell Research, 241, 316–323. 10.1006/excr.1998.4001 9637773

[acel13562-bib-0038] Matsumura, H. , Mohri, Y. , Binh, N. T. , Morinaga, H. , Fukuda, M. , Ito, M. , Kurata, S. , Hoeijmakers, J. , & Nishimura, E. K. (2016). Hair follicle aging is driven by transepidermal elimination of stem cells via COL17A1 proteolysis. Science, 351, aad4395. 10.1126/science.aad4395 26912707

[acel13562-bib-0071] McNeely, T. , Leone, M. , Yanai, H. , Beerman, I. (2020). DNA damage in aging, the stem cell perspective. Human Genetics, 139(3), 309–331. 10.1007/s00439-019-02047-z 31324975 PMC6980431

[acel13562-bib-0039] McWhir, J. , Selfridge, J. , Harrison, D. J. , Squires, S. , & Melton, D. W. (1993). Mice with DNA repair gene (ERCC‐1) deficiency have elevated levels of p53, liver nuclear abnormalities and die before weaning. Nature Genetics, 5, 217–224. 10.1038/ng1193-217 8275084

[acel13562-bib-0040] Milanese, C. , Bombardieri, C. R. , Sepe, S. , Barnhoorn, S. , Payán‐Goméz, C. , Caruso, D. , Audano, M. , Pedretti, S. , Vermeij, W. P. , Brandt, R. M. C. , Gyenis, A. , Wamelink, M. M. , de Wit, A. S. , Janssens, R. C. , Leen, R. , van Kuilenburg, A. B. P. , Mitro, N. , Hoeijmakers, J. H. J. , & Mastroberardino, P. G. (2019). DNA damage and transcription stress cause ATP‐mediated redesign of metabolism and potentiation of anti‐oxidant buffering. Nature Communications, 10, 4887. 10.1038/s41467-019-12640-5 PMC6814737 31653834

[acel13562-bib-0041] Muradian, K. , & Schachtschabel, D. O. (2001). The role of apoptosis in aging and age‐related disease: update. Zeitschrift Fur Gerontologie Und Geriatrie, 34, 441–446. 10.1007/s003910170015 11828881

[acel13562-bib-0042] Naipal, K. A. T. , Raams, A. , Bruens, S. T. , Brandsma, I. , Verkaik, N. S. , Jaspers, N. G. J. , Hoeijmakers, J. H. J. , van Leenders, G. J. L. H. , Pothof, J. , Kanaar, R. , Boormans, J. , & van Gent, D. C. (2015). Attenuated XPC expression is not associated with impaired DNA repair in bladder cancer. PLoS One, 10, e0126029. 10.1371/journal.pone.0126029 25927440 PMC4416023

[acel13562-bib-0043] Nalapareddy, K. , Nattamai, K. J. , Kumar, R. S. , Karns, R. , Wikenheiser‐Brokamp, K. A. , Sampson, L. L. , Mahe, M. M. , Sundaram, N. , Yacyshyn, M.‐B. , Yacyshyn, B. , Helmrath, M. A. , Zheng, Y. I. , & Geiger, H. (2017). Canonical Wnt Signaling Ameliorates Aging of Intestinal Stem Cells. Cell Reports, 18, 2608–2621. 10.1016/j.celrep.2017.02.056 28297666 PMC5987258

[acel13562-bib-0044] Navarro, S. , Meza, N. W. , Quintana‐Bustamante, O. , Casado, J. A. , Jacome, A. , McAllister, K. , Puerto, S. , Surralles, J. , Segovia, J. C. , & Bueren, J. A. (2006). Hematopoietic dysfunction in a mouse model for Fanconi anemia group D1. Molecular Therapy: The Journal of the American Society of Gene Therapy, 14, 525–535. 10.1016/j.ymthe.2006.05.018 16859999

[acel13562-bib-0045] Niedernhofer, L. J. , Garinis, G. A. , Raams, A. , Lalai, A. S. , Robinson, A. R. , Appeldoorn, E. , Odijk, H. , Oostendorp, R. , Ahmad, A. , van Leeuwen, W. , Theil, A. F. , Vermeulen, W. , van der Horst, G. T. J. , Meinecke, P. , Kleijer, W. J. , Vijg, J. , Jaspers, N. G. J. , & Hoeijmakers, J. H. J. (2006). A new progeroid syndrome reveals that genotoxic stress suppresses the somatotroph axis. Nature, 444, 1038–1043. 10.1038/nature05456 17183314

[acel13562-bib-0046] Niedernhofer, L. J. , Gurkar, A. U. , Wang, Y. , Vijg, J. , Hoeijmakers, J. H. J. , & Robbins, P. D. (2018). Nuclear Genomic Instability and Aging. Annual Review of Biochemistry, 87, 295–322. 10.1146/annurev-biochem-062917-012239 29925262

[acel13562-bib-0047] Niedernhofer, L. J. , Odijk, H. , Budzowska, M. , van Drunen, E. , Maas, A. , Theil, A. F. , de Wit, J. , Jaspers, N. G. J. , Beverloo, H. B. , Hoeijmakers, J. H. J. , & Kanaar, R. (2004). The structure‐specific endonuclease Ercc1‐Xpf is required to resolve DNA interstrand cross‐link‐induced double‐strand breaks. Molecular and Cellular Biology, 24, 5776–5787. 10.1128/MCB.24.13.5776-5787.2004 15199134 PMC480908

[acel13562-bib-0048] Nijnik, A. , Woodbine, L. , Marchetti, C. , Dawson, S. , Lambe, T. , Liu, C. , Rodrigues, N. P. , Crockford, T. L. , Cabuy, E. , Vindigni, A. , Enver, T. , Bell, J. I. , Slijepcevic, P. , Goodnow, C. C. , Jeggo, P. A. , & Cornall, R. J. (2007). DNA repair is limiting for haematopoietic stem cells during ageing. Nature, 447, 686–690. 10.1038/nature05875 17554302

[acel13562-bib-0049] Ogrodnik, M. , Miwa, S. , Tchkonia, T. , Tiniakos, D. , Wilson, C. L. , Lahat, A. , Day, C. P. , Burt, A. , Palmer, A. , Anstee, Q. M. , Grellscheid, S. N. , Hoeijmakers, J. H. J. , Barnhoorn, S. , Mann, D. A. , Bird, T. G. , Vermeij, W. P. , Kirkland, J. L. , Passos, J. F. , von Zglinicki, T. , & Jurk, D. (2017). Cellular senescence drives age‐dependent hepatic steatosis. Nature Communications, 8, 15691. 10.1038/ncomms15691 PMC5474745 28608850

[acel13562-bib-0050] Pollack, M. , Phaneuf, S. , Dirks, A. , & Leeuwenburgh, C. (2002). The role of apoptosis in the normal aging brain, skeletal muscle, and heart. Annals of the New York Academy of Sciences, 959, 93–107. 10.1111/j.1749-6632.2002.tb02086.x 11976189

[acel13562-bib-0051] Prasher, J. M. , Lalai, A. S. , Heijmans‐Antonissen, C. , Ploemacher, R. E. , Hoeijmakers, J. H. , Touw, I. P. , & Niedernhofer, L. J. (2005). Reduced hematopoietic reserves in DNA interstrand crosslink repair‐deficient Ercc1‐/‐ mice. The EMBO Journal, 24, 861–871. 10.1038/sj.emboj.7600542 15692571 PMC549615

[acel13562-bib-0052] Ritsma, L. , Ellenbroek, S. I. J. , Zomer, A. , Snippert, H. J. , de Sauvage, F. J. , Simons, B. D. , Clevers, H. , & van Rheenen, J. (2014). Intestinal crypt homeostasis revealed at single‐stem‐cell level by in vivo live imaging. Nature, 507, 362–365. 10.1038/nature12972 24531760 PMC3964820

[acel13562-bib-0053] Rossi, D. J. , Bryder, D. , Seita, J. , Nussenzweig, A. , Hoeijmakers, J. , & Weissman, I. L. (2007). Deficiencies in DNA damage repair limit the function of haematopoietic stem cells with age. Nature, 447, 725–729. 10.1038/nature05862 17554309

[acel13562-bib-0054] Rube, C. E. , Fricke, A. , Widmann, T. A. , Furst, T. , Madry, H. , Pfreundschuh, M. , & Rube, C. (2011). Accumulation of DNA damage in hematopoietic stem and progenitor cells during human aging. PLoS One, 6, e17487. 10.1371/journal.pone.0017487 21408175 PMC3049780

[acel13562-bib-0055] Sato, T. , Vries, R. G. , Snippert, H. J. , van de Wetering, M. , Barker, N. , Stange, D. E. , van Es, J. H. , Abo, A. , Kujala, P. , Peters, P. J. , & Clevers, H. (2009). Single Lgr5 stem cells build crypt‐villus structures in vitro without a mesenchymal niche. Nature, 459, 262–265. 10.1038/nature07935 19329995

[acel13562-bib-0056] Schumacher, B. , Pothof, J. , Vijg, J. , & Hoeijmakers, J. H. J. (2021). The central role of DNA damage in the ageing process. Nature, 592, 695–703. 10.1038/s41586-021-03307-7 33911272 PMC9844150

[acel13562-bib-0057] Shivdasani, R. A. (2014). Radiation redux: reserve intestinal stem cells miss the call to duty. Cell Stem Cell, 14, 135–136. 10.1016/j.stem.2014.01.015 24506878 PMC3955177

[acel13562-bib-0058] Sperka, T. , Song, Z. , Morita, Y. , Nalapareddy, K. , Guachalla, L. M. , Lechel, A. , Begus‐Nahrmann, Y. , Burkhalter, M. D. , Mach, M. , Schlaudraff, F. , Liss, B. , Ju, Z. , Speicher, M. R. , & Rudolph, K. L. (2011). Puma and p21 represent cooperating checkpoints limiting self‐renewal and chromosomal instability of somatic stem cells in response to telomere dysfunction. Nature Cell Biology, 14, 73–79. 10.1038/ncb2388 22138576

[acel13562-bib-0059] Steegenga, W. T. , Mischke, M. , Lute, C. , Boekschoten, M. V. , Lendvai, A. , Pruis, M. G. M. , Verkade, H. J. , van de Heijning, B. J. M. , Boekhorst, J. , Timmerman, H. M. , Plösch, T. , Müller, M. , & Hooiveld, G. J. E. J. (2017). Maternal exposure to a Western‐style diet causes differences in intestinal microbiota composition and gene expression of suckling mouse pups. Molecular Nutrition & Food Research, 61(1), 1600141. 10.1002/mnfr.201600141 PMC5215441 27129739

[acel13562-bib-0060] Tetteh, P. W. , Basak, O. , Farin, H. F. , Wiebrands, K. , Kretzschmar, K. , Begthel, H. , van den Born, M. , Korving, J. , de Sauvage, F. , van Es, J. H. , van Oudenaarden, A. , & Clevers, H. (2016). Replacement of Lost Lgr5‐Positive Stem Cells through Plasticity of Their Enterocyte‐Lineage Daughters. Cell Stem Cell, 18, 203–213. 10.1016/j.stem.2016.01.001 26831517

[acel13562-bib-0061] Tian, H. , Biehs, B. , Warming, S. , Leong, K. G. , Rangell, L. , Klein, O. D. , & de Sauvage, F. J. (2011). A reserve stem cell population in small intestine renders Lgr5‐positive cells dispensable. Nature, 478, 255–259. 10.1038/nature10408 21927002 PMC4251967

[acel13562-bib-0062] Tilstra, J. S. , Robinson, A. R. , Wang, J. , Gregg, S. Q. , Clauson, C. L. , Reay, D. P. , Nasto, L. A. , St Croix, C. M. , Usas, A. , Vo, N. et al (2012). NF‐kappaB inhibition delays DNA damage‐induced senescence and aging in mice. The Journal of Clinical Investigation, 122, 2601–2612. 22706308 10.1172/JCI45785 PMC3386805

[acel13562-bib-0063] van der Pluijm, I. , Garinis, G. A. , Brandt, R. M. , Gorgels, T. G. , Wijnhoven, S. W. , Diderich, K. E. , de Wit, J. , Mitchell, J. R. , van Oostrom, C. , Beems, R. , Niedernhofer, L. J. , Velasco, S. , Friedberg, E. C. , Tanaka, K. , van Steeg, H. , Hoeijmakers, J. H. J. , & van der Horst, G. T. J. (2007). Impaired genome maintenance suppresses the growth hormone–insulin‐like growth factor 1 axis in mice with Cockayne syndrome. PLoS Biology, 5, e2. 17326724 10.1371/journal.pbio.0050002 PMC1698505

[acel13562-bib-0064] Vermeij, W. P. , Dollé, M. E. T. , Reiling, E. , Jaarsma, D. , Payan‐Gomez, C. , Bombardieri, C. R. , Wu, H. , Roks, A. J. M. , Botter, S. M. , van der Eerden, B. C. , Youssef, S. A. , Kuiper, R. V. , Nagarajah, B. , van Oostrom, C. T. , Brandt, R. M. C. , Barnhoorn, S. , Imholz, S. , Pennings, J. L. A. , de Bruin, A. , … Hoeijmakers, J. H. J. (2016a). Restricted diet delays accelerated ageing and genomic stress in DNA‐repair‐deficient mice. Nature, 537, 427–431. 10.1038/nature19329 27556946 PMC5161687

[acel13562-bib-0065] Vermeij, W. P. , Hoeijmakers, J. H. , & Pothof, J. (2016b). Genome Integrity in Aging: Human Syndromes, Mouse Models, and Therapeutic Options. Annual Review of Pharmacology and Toxicology, 56, 427–445. 10.1146/annurev-pharmtox-010814-124316 26514200

[acel13562-bib-0066] Walter, D. , Lier, A. , Geiselhart, A. , Thalheimer, F. B. , Huntscha, S. , Sobotta, M. C. , Moehrle, B. , Brocks, D. , Bayindir, I. , Kaschutnig, P. , Muedder, K. , Klein, C. , Jauch, A. , Schroeder, T. , Geiger, H. , Dick, T. P. , Holland‐Letz, T. , Schmezer, P. , Lane, S. W. , … Milsom, M. D. (2015). Exit from dormancy provokes DNA‐damage‐induced attrition in haematopoietic stem cells. Nature, 520, 549–552. 10.1038/nature14131 25707806

[acel13562-bib-0067] Wang, J. , Clauson, C. L. , Robbins, P. D. , Niedernhofer, L. J. , & Wang, Y. (2012). The oxidative DNA lesions 8,5'‐cyclopurines accumulate with aging in a tissue‐specific manner. Aging Cell, 11, 714–716. 10.1111/j.1474-9726.2012.00828.x 22530741 PMC3399950

[acel13562-bib-0068] Weeda, G. , Donker, I. , de Wit, J. , Morreau, H. , Janssens, R. , Vissers, C. J. , Nigg, A. , van Steeg, H. , Bootsma, D. , & Hoeijmakers, J. H. (1997). Disruption of mouse ERCC1 results in a novel repair syndrome with growth failure, nuclear abnormalities and senescence. Current Biology, 7, 427–439. 10.1016/S0960-9822(06)00190-4 9197240

[acel13562-bib-0069] Yousefzadeh, M. , Henpita, C. , Vyas, R. , Soto‐Palma, C. , Robbins, P. , & Niedernhofer, L. (2021). DNA damage—how and why we age? eLife, 10, 10.7554/elife.62852 PMC7846274 33512317

[acel13562-bib-0070] Yousefzadeh, M. J. , Zhao, J. , Bukata, C. , Wade, E. A. , McGowan, S. J. , Angelini, L. A. , Bank, M. P. , Gurkar, A. U. , McGuckian, C. A. , Calubag, M. F. , Kato, J. I. , Burd, C. E. , Robbins, P. D. , & Niedernhofer, L. J. (2020). Tissue specificity of senescent cell accumulation during physiologic and accelerated aging of mice. Aging Cell, 19, e13094. 10.1111/acel.13094 31981461 PMC7059165

