# Supplementary figures and images for: Different responses to DNA damage determine ageing differences between organs
Source: Aging Cell. 2022 Mar 4;21(4):e13562. doi: 10.1111/acel.13562 (PMC9009128; doi:10.1111/acel.13562)

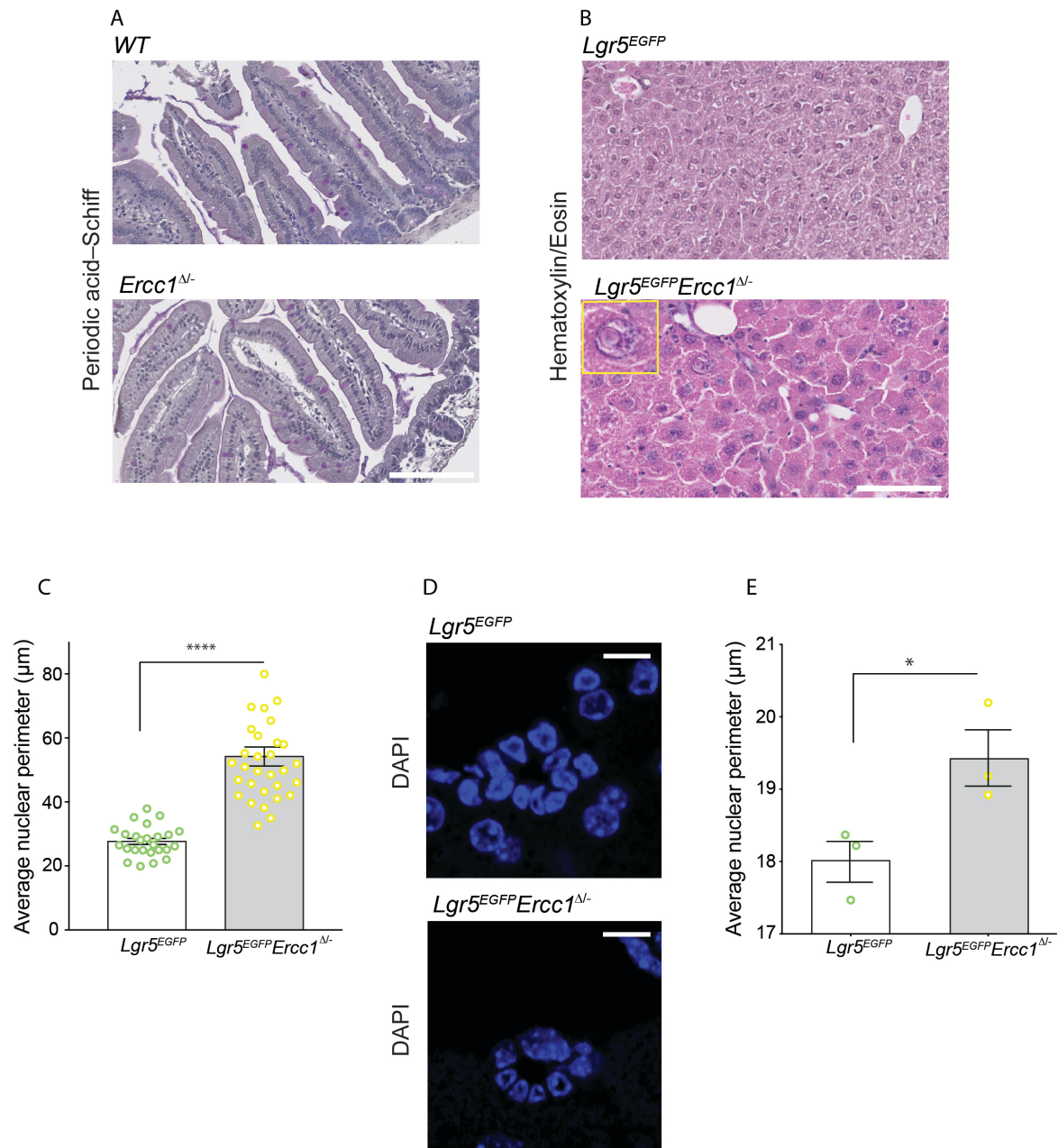

Sup. Fig. 1

Supplement: Supplementary file 1 — Fig S1 [file ACEL-21-e13562-s006.pdf]

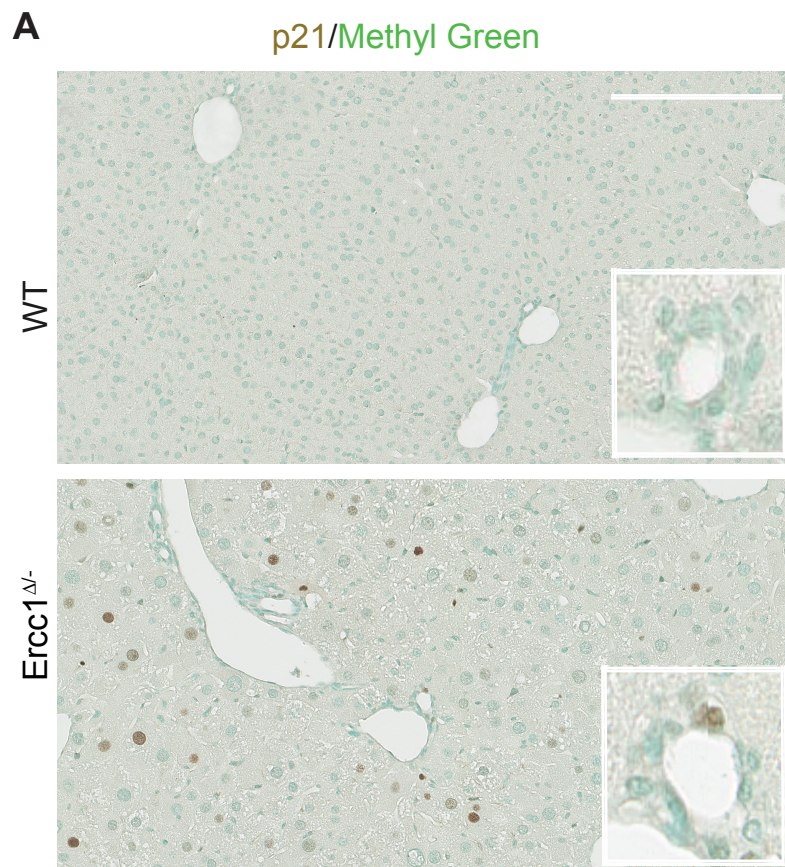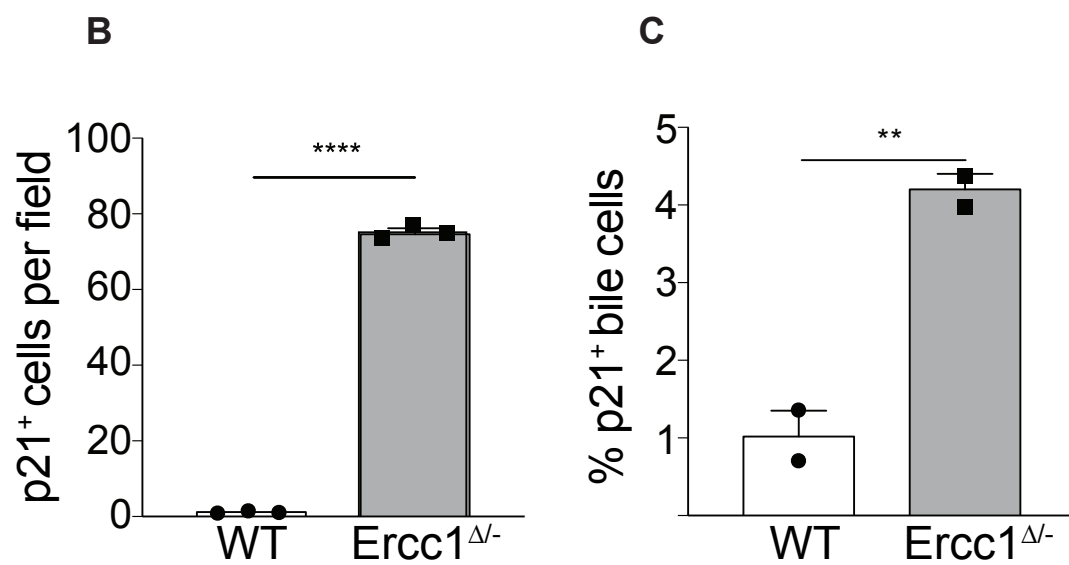

Supplement: Supplementary file 2 — Fig S2 [file ACEL-21-e13562-s002.pdf]

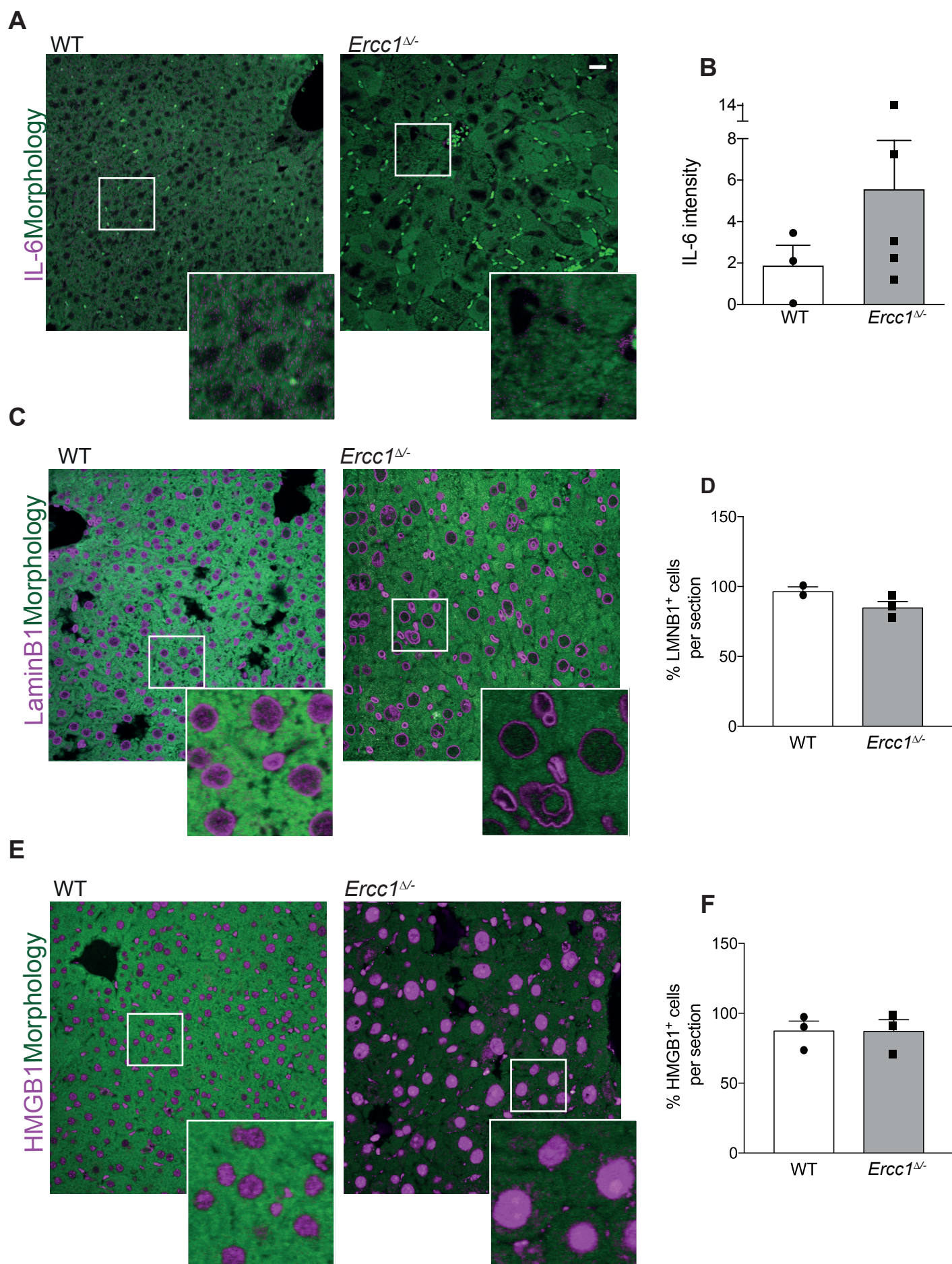

Supplementary Figure 3

Supplement: Supplementary file 3 — Fig S3 [file ACEL-21-e13562-s007.pdf]

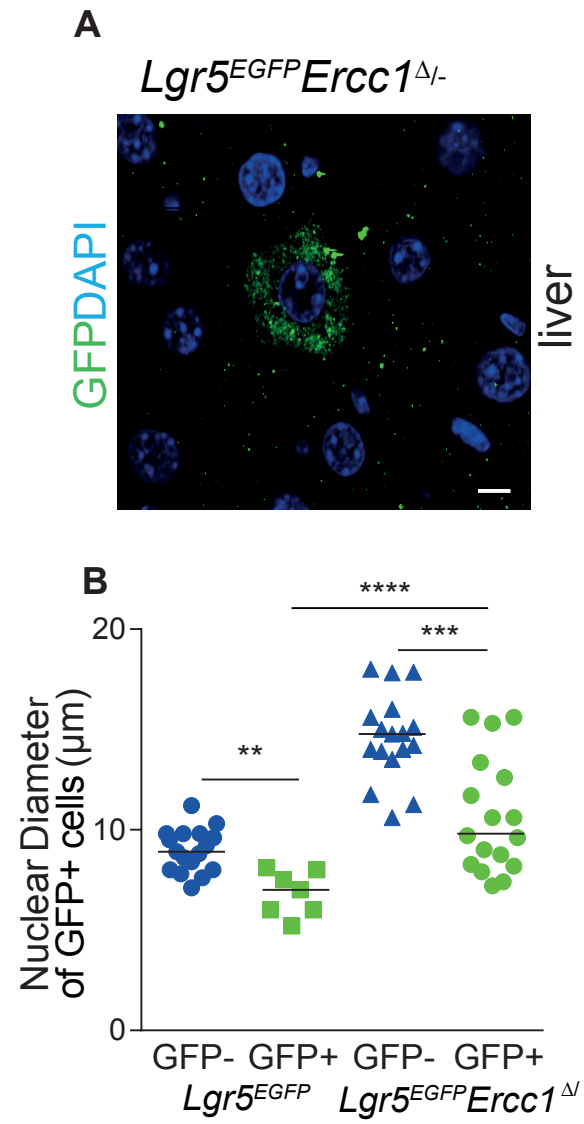

Supplement: Supplementary file 4 — Fig S4 [file ACEL-21-e13562-s004.pdf]

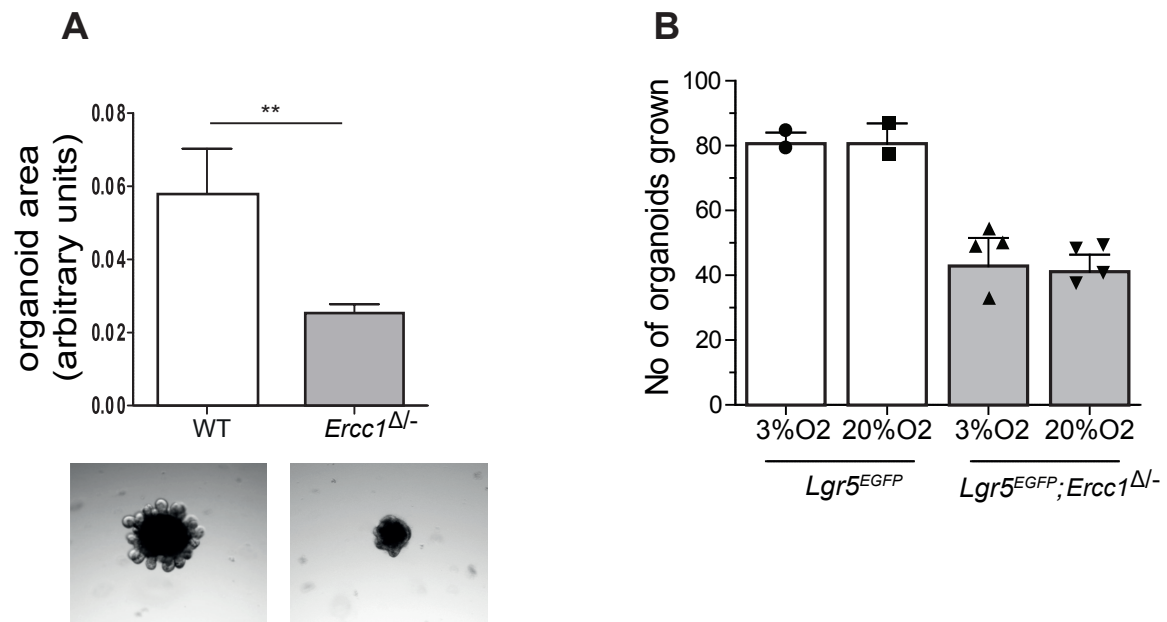

Supplementary Figure 5

Supplement: Supplementary file 5 — Fig S5 [file ACEL-21-e13562-s005.pdf]

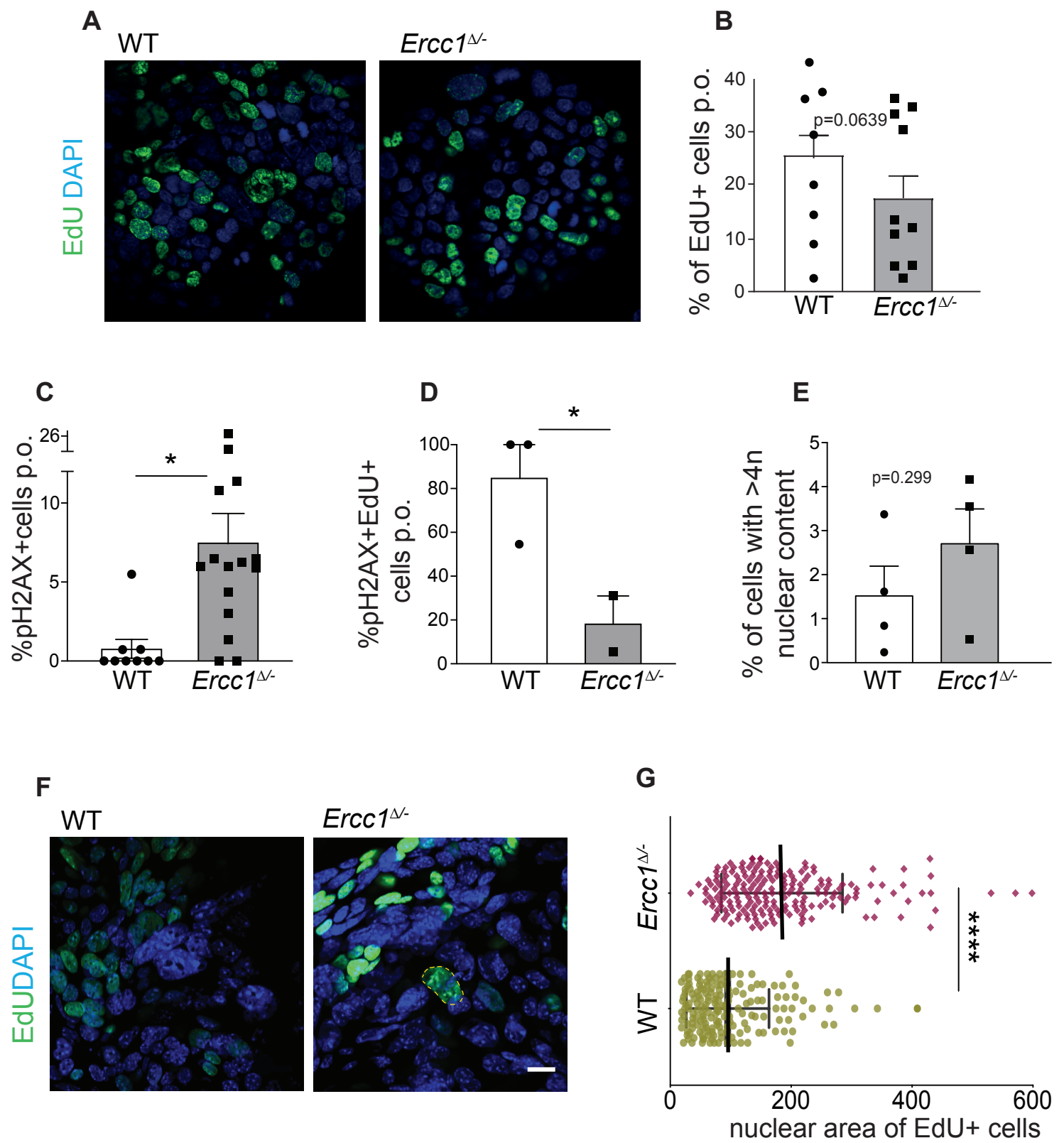

Supplementary Figure 6

Supplement: Supplementary file 6 — Fig S6 [file ACEL-21-e13562-s003.pdf]
