# Supplementary material for: Different responses to DNA damage determine ageing differences between organs
Source: Aging Cell. 2022 Mar 4;21(4):e13562. doi: 10.1111/acel.13562 (PMC9009128; doi:10.1111/acel.13562)
Supplement: Supplementary file 7 — Supplementary Material [file ACEL-21-e13562-s001.docx]

**SUPPLEMENTARY INFORMATION**

**SUPPLEMENTARY METHODS**

**Immunohistochemistry and Immunocytochemistry**

Intestinal and liver tissue were fixed in 10% buffered formalin overnight, embedded in paraffin and sectioned. 4 μm sections were de-paraffinized and rehydrated according to standard procedures. Hematoxylin and Eosin staining, as well as Periodic acid–Schiff and Methyl Green (counter)staining - where appropriate - were performed following standard protocols. For immunohistochemistry, antigen retrieval was performed using 0.01M sodium citrate solution pH 6.0 (in a microwave, ~700W for 20min). For p21 DAB stainings antigen retrieval was performed by incubation of the tissue sections in 10 mM Tris-base, 1 mM EDTA, 0.05% Tween 20, pH 9.0 solution. Subsequently, slides with tissue sections were incubated with primary antibody in TBS supplemented with 0.1% TritonX100 and 10% Normal Horse Serum, overnight at 4^0^ C. Antibodies used: anti-ki67 (Millipore, Cat# AB9260), anti-p21Cip (BD Transduction Laboratories, Cat#610234), anti-EGFP (Abcam, Cat#ab13970), anti-Lamin B1 (Abcam, Cat#ab16048), anti-HMGB1 (Abcam Cat#ab77302), anti-γH2AX (Millipore, Cat#05336), anti-Cytochrome c (BD Pharmigen, Cat#556432), anti-Caspase 3 (Cell signaling, Cat#9661). Stainings were visualized with appropriate secondary antibodies labeled with Alexa488/555 (Life Technologies). DAB stainings were developed using biotin-labeled secondary antibodies and Vectastain ABC Elite kit.

**Microscopy**

Immunofluorescent images were acquired on a Zeiss LSM700 confocal microscope. Pictures from Immunoperoxidase and Hematoxylin/Eosin-stained sections were acquired using Nanozoomer 2.0-HT. Images were analysed using Fiji software as well as Hamamatsu NDP viewer software. Acquisition of images from organoid cultures was done using a Leitz Aristoplan phase contrast microscope.

**FACS and flow cytometric analysis**

Small intestinal crypts were isolated as previously described ([Huch et al., 2013](#_ENREF_1); [Sato et al., 2009](#_ENREF_2)) and subsequently incubated in TrypLE solution in order to obtain single cell suspensions. Suspensions were filtered with a 40 μm strainer and EGFP-expressing cells were analysed on a FACSAria III cell sorter (BD).

Liver organoid cultures were harvested from matrigel by incubation in TrypLE solution at 37^0^C for approximatetly 15 min and gentle pipetting until single cell suspensions were obtained. Concominantly, cells were resuspended in 0.998 mL PBS supplemented with 0,1% Triton X100. 1μL Hoechst 33342 (10mg/mL, Life Technologies) was added and the cells were incubated at 37^0^C for 15 minutes. Cells were resuspended in 4% PFA and analyzed for incorporated Hoechst using a BD LSRFortessa flow cytometer. Analysis of experiments was performed using Flow-jo software vX.0.7 (Tree Star Inc.).

**SUPPLEMENTARY FIGURE LEGENDS**

Supplementary Figure 1

**Histological analysis of *Ercc1^Δ/-^* intestinal and liver tissue**

(A) Intestinal mucus-forming functionality unchanged in *Ercc1^Δ/-^* progeroid mice. Representative pictures of paraffin-embedded intestinal tissue from a 15-week-old *Ercc1^Δ/-^* mouse and corresponding wild type control, Periodic acid–Schiff stained and hematoxylin as counterstaining. Scale bars represent 100 μm.

(A) Liver pathology similar to ageing in *Lgr5^EGFP^Ercc1^Δ/-^* progeroid mice. Representative picture of paraffin-embedded liver tissue from a 15-week-old *Lgr5^EGFP^Ercc1^Δ/-^* mouse and corresponding *Lgr5^EGFP^* control, stained with hematoxylin and eosin dyes. The inset demonstrates a polyploid hepatocyte nucleus with cytoplasmic inclusions. Scale bars represent 100 μm.

(B) Quantitation of the average perimeter of nuclei in paraffin-embedded liver sections from 15-week-old *Lgr5^EGFP^Ercc1^Δ/-^*  and *Lgr5^EGFP^* control mice (at least 3 slices were quantified for a total of 3 mice per genotype).

(C) Images of bile cell nuclei from 15-week-old mice of the indicated genotypes stained with DAPI. Scale bars represent 10 μm

(D) Quantitation of the average perimeter of bile cell nuclei from paraffin-embedded liver sections from 15-week-old mice of the indicated genotypes (n=3 mice per genotype).

Data represent mean ± S.E.M. *p<0,05, ****p<0,0001

Supplementary Figure 2

**Assessment of cell cycle arrest in 15-week-old *Ercc1^Δ/-^* liver tissue.**

(A) Upregulation of p21 in 15-week-old *Ercc1^Δ/-^* liver. Representative images of 3,3'-diaminobenzidine (DAB) immunohistochemical staining against p21 on paraffin-embedded liver tissue from the indicated genotypes. Counterstaining was performed using Methyl green dye. Scale bars represent 200 μm.

(B and C) Quantitation of p21-positive cell populations from liver tissue immunohistochemical stainings represented in (a) (n=3 mice per genotype for quantitation of total p21 positive cells and n=2 mice per group for quantitation of bile cell population).

Data represent mean ± S.E.M. **p<0,01, ****p<0,0001

Supplementary Figure 3

**Evaluation of senescence markers in the liver of 15-week-old *Ercc1^Δ/-^* mice**

(A) Representative images of immunofluorescence staining against IL-6 (purple) on paraffin-embedded liver sections from 15-week-old mice of the indicated genotypes. Scale bar represents 10 μm.

(B) Quantitation of the cell nuclei that are positive for IL-6 on stained liver sections from 15-week-old mice of the indicated genotypes, p=0,148 (n=3 and n=5 for WT and mutant mice respectively).

(C) Representative images of immunofluorescence staining against Lamin B1 (purple) on paraffin-embedded liver sections from 15-week-old mice of the indicated genotypes.

(D) Quantitation of the cell nuclei that are positive for Lamin B1 on stained liver sections from 15-week-old mice of the indicated genotypes, p=0,2 (n=2-3 mice per genotype).

(E) Representative images of immunofluorescence staining against HMGB1 (red) on paraffin-embedded liver sections from 15-week-old mice of the indicated genotypes.

(F) Quantitation of the fluorescence intensity (expressed in arbitrary units) of HMGB1 immunosignal on stained liver sections from 15-week-old mice of the indicated genotypes, p=0,98 (n=3 mice per genotype).

Data represent mean ± S.E.M.

Supplementary Figure 4

**Evaluation of liver stem cell population in 15-week-old *Ercc1^Δ/-^* mice**

(A) Detection of polyploid EGFP+ hepatocytes in 15-week-old *Lgr5^EGFP^Ercc1^Δ/-^* liver tissue. Representative picture of such a nucleus from paraffin-embedded tissue sections stained for GFP. Scale bar represents 10μm.

(B) Graph depicting the average nuclear diameter of EGFP (Lgr5+) expressing relative to non-expressing (EGFP-) hepatocytes of 15-week-old *Lgr5^EGFP^Ercc1^Δ/-^* and *Lgr5^EGFP^* control mice. Values of individual hepatocytes from a total of 3 mice per genotype are represented in the graph). Nuclear diameter was quantitated from immunofluorescently labeled tissue sections as in (a) using Fiji software and is expressed in arbitrary units. Note the significant difference between EGFP+ and EGFP- cells in both genotypes. Note also the increase in nuclear size of EGFP+ cells in progeroid *Ercc1^Δ/-^* liver.

Bars indicate group medians. **p<0,01, ***p<0,001, ****p<0,0001.

Supplementary Figure 5

**SI and LSC organoid phenotypes are not due to oxidative stress during culture**

(A) Quantitation of the average organoid size of the indicated genotypes (n=3 independent cultures from different mice per group) after 9 days in culture. Freshly-isolated crypts from 15-week-old mice were cultured under low oxygen conditions (3% O_2_). Below the graph representative images of organoids from the respective experiment at day 9 of culture.

(B) Quantitation of liver organoids grown after culturing single LSCs suspension derived from a primary culture of bile duct cells from 15-week-old mice of the indicated genotypes under high (20%) and low (3%) O_2_ conditions without or with antioxidant supplementation, respectively (p=0,0013 for low and p=0,005 for high oxidative conditions). Counting was performed after 7 days in culture. (n=2 and n=3 mice for WT and mutant, respectively).

Data represent mean ± S.E.M. **p<0,01.

Supplementary Figure 6

**Proliferative index and nuclear content of *Ercc1^Δ/-^* liver organoids.**

(A) Representative images of incorporated EdU immunofluorescence in liver organoids grown from 15-week-old mice of the indicated genotypes.

(B) Quantitation of the percentage of replicating cells per *Ercc1^Δ/-^* and corresponding WT control organoids in *in vitro* cultures (individual dots represent values of separate organoid cultures from 3 mice per genotype in total). Note the marginally reduced fraction of *Ercc1^Δ/-^* liver stem cells (LSCs) undergoing replication.

(C) Detection of phosphorylated H2AX in *Ercc1^Δ/-^* LSC organoids. Quantitation of the percentage of γH2AX-positive LSCs in wt and *Ercc1^Δ/-^* organoids from immunofluorescently labeled samples, derived from 15-week-old mice of the indicated genotypes. Data represent mean with SD of separate cultures of organoid clones derived from 3 different mice.

(D) Quantitation of the percentage of EdU+ LSCs that show γH2AX immunosignal in liver organoids derived from 15-week-old mice of the indicated genotypes (n=3 and n=2 mice per indicated genotype, respectively).

(E) Quantitation by flow cytometry for Hoechst fluorescence, of DNA content of WT and *Ercc1^Δ/-^* liver organoid single cell suspensions. The measurements of two individual clonal cultures grown each single mouse (2 mice in total) are collectively plotted. p=0.2996.

(F) Increased polyploidy index in *Ercc1^Δ/-^* LSCs. Representative images of incorporated EdU immunofluorescence in organoid LSCs 48 hours after a short, half-hour pulse of EdU. Scale bar represents 10 μm. Note the presence of enlarged EdU-positive nuclei (in the picture encircled with a dashed line) in *Ercc1^Δ/-^* LSCs.

(G) Quantification of the nuclear area of EdU-positive nuclei in organoid LSC cultures derived from 15-week-old mice *Ercc1^Δ/-^* mice and corresponding controls, 48 hours after a half-hour pulse of EdU. The nuclear area was measured with Fiji software and is presented in arbitrary units. Plotted are the values of approximately equal number of organoid nuclei per mouse (3 mice per genotype). Bars indicate group medians and SD.

Data represent mean ± S.E.M. unless otherwise specified. *p<0,05, ****p<0,0001.

**SUPPLEMENTARY REFERENCES**

Huch, M., Dorrell, C., Boj, S.F., van Es, J.H., Li, V.S., van de Wetering, M., Sato, T., Hamer, K., Sasaki, N., Finegold, M.J.*, et al.* (2013). In vitro expansion of single Lgr5+ liver stem cells induced by Wnt-driven regeneration. Nature *494*, 247-250.

Sato, T., Vries, R.G., Snippert, H.J., van de Wetering, M., Barker, N., Stange, D.E., van Es, J.H., Abo, A., Kujala, P., Peters, P.J.*, et al.* (2009). Single Lgr5 stem cells build crypt-villus structures in vitro without a mesenchymal niche. Nature *459*, 262-265.
